# Novel Insights into the Roles of Bcl-2 Homolog Nr-13 (vNr-13) Encoded by Herpesvirus of Turkeys in the Virus Replication Cycle, Mitochondrial Networks, and Apoptosis Inhibition

**DOI:** 10.1128/JVI.02049-19

**Published:** 2020-05-04

**Authors:** Vishwanatha R. A. P. Reddy, Yashar Sadigh, Na Tang, Yongxiu Yao, Venugopal Nair

**Affiliations:** a The Pirbright Institute, Woking, United Kingdom; b Jenner Institute, University of Oxford, Oxford, United Kingdom; c Department of Zoology, University of Oxford, Oxford, United Kingdom; University of Southern California

**Keywords:** wild-type HVT, vNr-13, HVT-Δ*vNr-13*, Bcl-2, CRISPR, apoptosis, Bcl-2 family

## Abstract

The Bcl-2 (B cell lymphoma 2)-related protein Nr-13 plays a major role in the regulation of cell death in developing avian B cells. With over 65% sequence similarity to the chicken Nr-13, herpesvirus of turkeys (HVT) vNr-13, encoded by the HVT079 and HVT096 genes, is the first known alphaherpesvirus-encoded Bcl-2 homolog. HVT-infected cells were reported to be relatively more resistant to serum starvation, suggested that vNr-13 could be involved in protecting the cells. Here, we describe CRISPR/Cas9-based editing of exon 1 of the HVT079 and HVT096 genes from the HVT genome to generate the mutant HVT-Δ*vNr-13* to gain insights into its functional roles. Overall, wild-type HVT and HVT-Δ*vNr-13* showed similar growth kinetics; however, at early time points, HVT-Δ*vNr-13* showed 1.3- to 1.7-fold-lower growth of cell-associated virus and 3- to 6.2-fold-lower growth of cell-free virus. In transfected cells, HVT vNr-13 showed a mainly diffuse cytoplasmic distribution with faint nuclear staining. Further, vNr-13 localized to the mitochondria and endoplasmic reticulum (ER) and disrupted mitochondrial network morphology in the transfected cells. In the wild-type HVT-infected cells, *vNr-13* expression appeared to be directly involved in the disruption of the mitochondrial network, as the mitochondrial network morphology was substantially restored in the HVT-Δ*vNr-13*-infected cells. IncuCyte S3 real-time apoptosis monitoring demonstrated that vNr-13 is unequivocally involved in the apoptosis inhibition, and it is associated with an increase of PFU, especially under serum-free conditions in the later stages of the viral replication cycle. Furthermore, HVT blocks apoptosis in infected cells but activates apoptosis in noninfected bystander cells.

**IMPORTANCE** B cell lymphoma 2 (Bcl-2) family proteins play important roles in regulating apoptosis during homeostasis, tissue development, and infectious diseases. Several viruses encode homologs of cellular Bcl-2-proteins (vBcl-2) to inhibit apoptosis, which enable them to replicate and persist in the infected cells and to evade/modulate the immune response of the host. Herpesvirus of turkeys (HVT) is a nonpathogenic alphaherpesvirus of turkeys and chickens that is widely used as a live vaccine against Marek’s disease and as recombinant vaccine viral vectors for protecting against multiple avian diseases. Identical copies of the HVT genes HVT079 and HVT096 encode the Bcl-2 homolog vNr-13. While previous studies have identified the potential ability of vNr-13 in inhibiting apoptosis induced by serum deprivation, there have been no detailed investigations on the functions of vNr-13. Using CRISPR/Cas9-based ablation of the *vNr-13* gene, we demonstrated the roles of HVT vNr-13 in early stages of the viral replication cycle, mitochondrial morphology disruption, and apoptosis inhibition in later stages of viral replication.

## Abstract (precis)

B cell lymphoma 2 (Bcl-2) family proteins play important roles in regulating apoptosis during homeostasis, tissue development, and infectious diseases. Several viruses encode homologs of cellular Bcl-2-proteins (vBcl-2) to inhibit apoptosis, which enable them to replicate and persist in the infected cells and to evade/modulate the immune response of the host. Herpesvirus of turkeys (HVT) is a nonpathogenic alphaherpesvirus of turkeys and chickens that is widely used as a live vaccine against Marek’s disease and as recombinant vaccine viral vectors for protecting against multiple avian diseases. Identical copies of the HVT genes HVT079 and HVT096 encode the Bcl-2 homolog vNr-13. While previous studies have identified the potential ability of vNr-13 in inhibiting apoptosis induced by serum deprivation, there have been no detailed investigations on the functions of vNr-13. Using CRISPR/Cas9-based ablation of the *vNr-13* gene, we demonstrated the roles of HVT vNr-13 in early stages of the viral replication cycle, mitochondrial morphology disruption, and apoptosis inhibition in later stages of viral replication.

## INTRODUCTION

Herpesvirus of turkeys (HVT), also known as Meleagrid herpesvirus 1 (MeHV1), belongs to the genus *Mardivirus* in the subfamily *Alphaherpesvirinae* of the family *Herpesviridae*. Originally isolated from domestic turkeys (1, 2), HVT is successfully used as a live viral vaccine against Marek’s disease (MD), a rapid-onset lymphomatous and paralytic disease caused by Marek’s disease virus 1 (MDV-1) in poultry (3). HVT is also used as a very successful recombinant vaccine vector to protect against multiple avian diseases.

Apoptosis, or programmed cell death, is a natural tissue homeostasis process that eliminates damaged, dangerous, and infected cells during pathogen invasion or unwanted cells during embryonic development (4). Bcl-2 (B cell lymphoma 2) family proteins play a principal role in the regulation of cell survival or death decisions and control mitochondrion-related cell death (4, 5). Currently, more than 20 Bcl-2 family proteins have been characterized, based on the presence of conserved Bcl-2 homology (BH) domains (4). Structurally and phylogenetically, the Bcl-2 family proteins are separated into two major groups. One group in the Bcl-2 family consists of proteins with a globular α-helical fold structure, i.e., a Bcl-2 fold with up to four BH domains with prosurvival (Bcl-2, Bcl-w, Bcl-xL, Mcl-1, Bcl-B, and A1) or proapoptotic (Bok, Bax, and Bak) proteins. Another group in the Bcl-2 family consists of distantly related intrinsically disordered proteins with only a simple BH3 domain (Bim, Bmf, Bad, Bid, Bik, Noxa, Hrk, and Puma), which antagonize or activate the Bcl-2 fold proteins of the other group. The prosurvival Bcl-2 fold proteins (Bcl-2, Bcl-w, Bcl-xL, Mcl-1, Bcl-B, and A1) inhibit the proapoptotic proteins of the BH3-only group (i.e., Bax, Bok, and Bak). The network of interactions and interplay between prosurvival and proapoptotic Bcl-2 proteins determines the fate of cells, and dysregulation of these interactions leads to disease conditions (4).

Many viruses have exploited the Bcl-2 pathway to modulate the cell environment, mainly to prevent premature death of the host cell to support virus replication. Several large DNA viruses, particularly lymphotropic gammaherpesviruses, encode Bcl-2-like proteins (vBcl-2) to evade apoptosis of cells to support virus replication (6, 7). Sequence analysis of the HVT genome revealed that two copies of the HVT genes, HVT079 and HVT096, potentially encoded a close homolog of the Bcl-2 protein Nr-13, the first and only example of an alphaherpesvirus-encoded Bcl-2 homolog (8–11).

Mitochondria are dynamic organelles that are required for several cellular processes, such as maintenance of cellular homeostasis, apoptosis regulation, and other signaling pathways. The dynamics of mitochondrial network pathophysiology have been broadly studied in many neurodegenerative diseases, such as Parkinson’s disease (PD), Alzheimer’s disease (AD), and Huntington disease (HD) (12). Several viruses and bacteria have been also reported to change the dynamics and function of mitochondrial networks (13–16). Specifically, hepatitis B virus-encoded X protein, associated with cell death, and human cytomegalovirus-encoded UL37x1 protein, associated with apoptosis inhibition, were reported to play roles in the disruption of mitochondrial networks (13, 17). Furthermore, alphaherpesviruses have been shown to play important roles in the mitochondrial distribution and morphology of infected cells (14, 16, 18), although there are yet no reports on the role of vBcl-2 proteins in modulating the mitochondrial network morphology.

While previous experiments using serum starvation or treatment with drugs such as staurosporine (SP) and thapsigargin (TG) have demonstrated the antiapoptotic properties of vNr-13 (8), its direct functional role in the replication of HVT or the effects on mitochondrial network morphology and interaction with the host cell have not yet been examined. The CRISPR/Cas9-based gene editing approach has been exploited in many fields of biological and clinical research (19–21). A number of previous studies have shown that CRISPR/Cas9 technology is an efficient and rapid approach for the manipulation of viral genomes compared to other recombination-based methods (22), as it allows direct functional analysis of genes without selection markers. Here, we report the application of CRISPR/Cas9-based editing to generate the HVT-Δ*vNr-13* deletion mutant virus to examine the functions of the vNr-13 homolog. Direct comparison of the infection dynamics of the wild-type and HVT-Δ*vNr-13* deletion mutant viruses was used to gain functional insights into its role in virus replication, mitochondrial network morphology, and regulation of apoptosis.

## RESULTS

### Sequence alignment of HVT vNr-13 and Bcl-2 orthologs.

**FIG 1:**
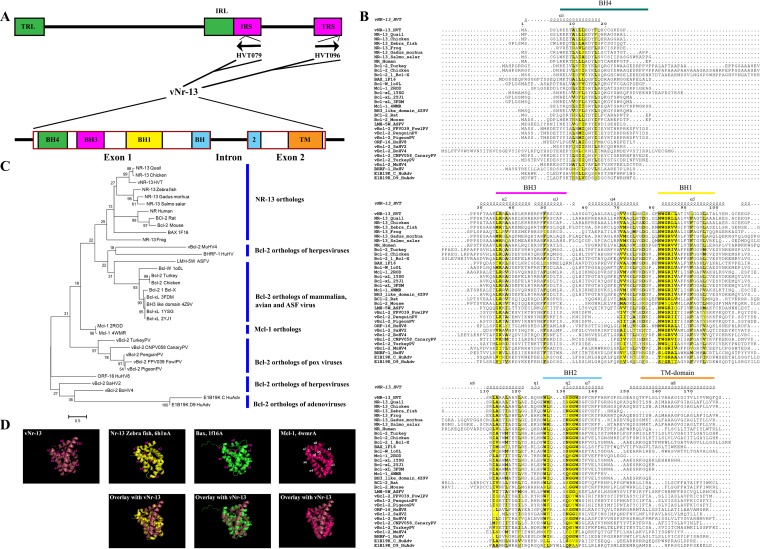
HVT vNr-13 structural analysis and sequence alignments with viral and cellular Bcl-2 orthologs of various mammalian and avian species. (A) Two identical copies of *vNr-13*, HVT079 in the reverse direction and HVT096 in the forward direction, are present in inverted repeat sequences (IRS) and terminal repeat sequences (TRS) of the HVT genome, respectively. HVT *vNr-13* has two exons and one intron. Bcl-2 homology domains (BH4, BH3, BH1, and BH2) and a transmembrane (TM) domain are present in exons in the 5′ to 3′ direction of the gene. (B) Qualitative analysis of sequence identity and similarity was performed using the ESPript 3.0 online tool. Helices 1 to 8 (α1 to α8) are shown above the sequence along with helix 9 of the TM domain, based on the vNr-13 predicted three-dimensional (3D) structural model. Strictly conserved residues are boxed in black on a yellow background. BH domains (BH4, BH3, BH1, and BH2) and the TM domain are marked above the sequence in the 5′ to 3′ direction. (C) Maximum-likelihood phylogenetic trees based on amino acid sequences of HVT vNr-13 in relation to other mammalian and viral orthologs. Bootstrap values of 1,000 replicates were assigned for the analysis. HVT vNr-13 was grouped separately with other Nr-13 orthologs. (D) Similar 3D homology of vNr-13 with zebrafish Nr-13, Bax, and Mcl-1, represented as a cartoon structural diagram. The 3D structures of vNr-13 (raspberry red), zebrafish Nr-13 (yellow), Bax (green), and Mcl-1 (magenta/hot pink) have identical orientations with eight α-helices, labeled α1 to α8. TM, transmembrane domain of vNr-13 and Mcl-1. All views are same as for vNr-13.

It was previously shown by Afonso et al. (9) and Aouacheria et al. (8) that the HVT genome sequence carries two identical *vNr-13* open reading frames (ORFs), HVT079 (positions 124354 to 125510) in the reverse direction and HVT096 (positions 157086 to 158242) in the forward direction, in the inverted repeat short (IRS) and terminal repeat short (TRS) sequences, respectively (Fig. 1A). Both the HVT079 and HVT096 copies of *vNr-13* have two exons and one intron, and their coding sequences contain 540 nucleotides, encoding 179-amino-acid proteins (8, 9). Afonso et al. (9) have reported the truncated isoform of vNr-13 from the N-terminal moiety encoded by the first 84 nucleotides of the introns to a 162-amino-acid protein, but the translated protein sequences of the introns were not available in the online database. It could be that *vNr-13* ORFs encoding identical 179-amino-acid proteins are present in the HVT genome, but the success of their identification depends on the ORF prediction software that was used. Indeed, this was confirmed also by other reports (8, 23). Furthermore, we have confirmed the full-length sequence of the *vNr-13* transcript from chicken embryo fibroblasts (CEFs) infected with HVT FC126 virus stocks.

Previous studies have reported that the vNr-13 sequence exhibits more than 63.7% identity with chicken Nr-13 (8–10). However, recently many other Bcl-2 orthologs of cellular and viral origin have been characterized, and their identity and/or similarity with vNr-13 is sparse (4, 5). Hence, we have extended our study to evaluate 34 cellular and viral Bcl-2 orthologs to examine the sequence identity and/or similarity with vNr-13. Multiple-sequence alignments of 34 cellular and viral Bcl-2 orthologs revealed that vNr-13 has highest sequence identity/similarity with Nr-13 of chickens (64.40%/66.66%), quail (62.71%/65.53%), zebrafish (38.81%/43.42%), and frog (30.00%/40.58%) and lowest homology with viral Bcl-2 sequences of penguin (09.14%/19.42%) and pigeon (09.14%/20.57%) pox viruses, murine herpesvirus 4 (09.35%/16.95%), and human adenovirus C (09.71%/16.00%). The ESPript 3.0 server (24) was used to determine identities and similarities among the orthologs. The vNr-13 amino acid sequence was reported to have all of the BH domains (BH4, BH3, BH1, and BH2) and the C-terminal transmembrane domain in the 5′-to-3′ direction (8–10, 23). All these BH domains are highly conserved among the Bcl-2 family proteins. In BH4, LL was conserved in Nr-13 from quail, chicken, and human and in Diva/Boo Bcl-2 from rat and mouse (10, 25). In BH4, YF was conserved in Nr-13 from quail and chicken and canary pox virus Bcl-2. In BH3, LR, A, and F were conserved among Nr-13 and Bcl-2 orthologs of most species. In BH1, NWGR was highly conserved among Nr-13 and Bcl-2 orthologs, except in human adenoviruses and murine herpesviruses. In BH2, GGW was strongly conserved among Nr-13 and Bcl-2 orthologs, except in human adenoviruses, murine herpesviruses, and pox viruses. Between BH1 and BH2, LA and WL were highly conserved amino acids among Nr-13 orthologs (Fig. 1B). A maximum-likelihood phylogenetic tree was constructed for the amino acid sequences of 32 cellular and viral Bcl-2 orthologs using the Le-Gascuel model with gamma distribution and invariant sites (26). The phylogenetic analysis of 32 cellular and viral Bcl-2 orthologs demonstrated that the Nr-13 proteins of chicken, quail, frog, zebrafish, and human and Bcl-2 of rat and mouse were clustered more closely with vNr-13 (Fig. 1C).

### HVT vNr-13 adopts a “Bcl-2-like” fold.

A three-dimensional (3D) homology model of vNr-13 was built by using the I-TASSER online tool (https://zhanglab.ccmb.med.umich.edu/I-TASSER/). For vNr-13, the predicted top model 1 had a confidence score (C score) of 0.25, an estimated template modeling (TM) score of 0.75 ± 0.11, and a root mean square deviation (RMSD) of 4.6 ± 3.0 Å. Eight α-helices were observed in the predicted vNr-13 model, which form a globular bundle, a topology characteristic of prosurvival Bcl-2 proteins (Fig. 1D). Based on protein function prediction by COFACTOR and COACH of the I-TASSER online server (27), the predicted vNr-13 model was the closest to other Bcl-2 family prosurvival proteins such as Bcl-xL (2yj1C) (C score of 0.51) and Mcl-1 (4wmrA) (C score of 0.20). The COFACTOR and COACH servers of I-TASSER are commonly used to predict 3D models of proteins based on protein functions (27). According to the structural similarity simulation, TM-align of I-TASSER, the predicted vNr-13 structure was found to have highest similarity with the proapoptotic protein Bax (1f16A) (highest TM score of 0.779 and RMSD of 2.44) and prosurvival protein Nr-13 (6h1nA) (TM score of 0.754 and RMSD of 1.33) of zebrafish. A superimposition of vNr-13 with Bax (1f16A), NR-13 zebrafish (6h1nA), and Mcl-1 (4wmrA) showed a good overlay between the structures (Fig. 1D).

### Analysis of the *in vitro* expression of HVT *vNr-13*.

**FIG 2:**
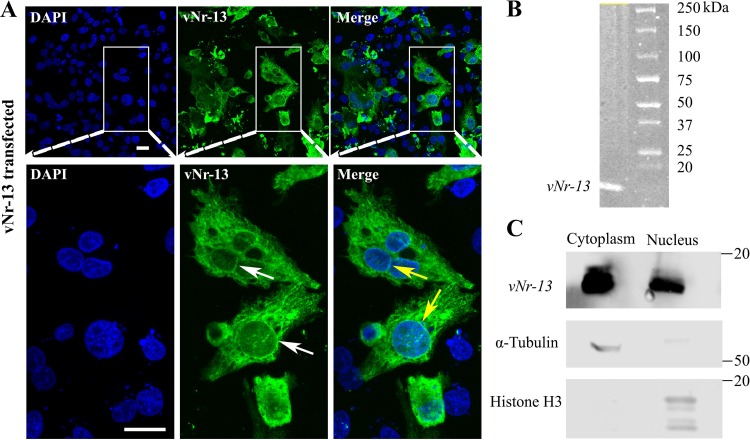
*In vitro* characterization of HVT vNr-13 by confocal microscopy and Western blotting. (A) Representative confocal image illustrating vNr-13 in DF-1 cells. Immunofluorescence staining was carried out in DF-1 cells transfected with expression construct pcDNA3.1-*vNr-13*. Cells were fixed after 48 h and stained with monoclonal F878 EG2 antibody (green). HVT vNr-13-transfected cells appeared to show diffuse staining throughout the cytoplasm and a relatively faint nuclear staining with apparent labeling of the nuclear envelope (white arrows). The cyan blue color shows vNr-13 colocalization with the nuclear envelope or membrane of the nuclei (yellow arrows). Scale bars, 20 μm. (B) Western blot analysis demonstrates that the F878 EG2 antibody specifically recognizes an ∼19-kDa band of HVT vNr-13. (C) Representative Western blot images of cytosolic and nuclear fractions. The purity of subcellular fractions was assayed with anti-α-tubulin or anti-histone H3 antibodies for the cytosolic or nuclear fraction, respectively.

**FIG 3:**
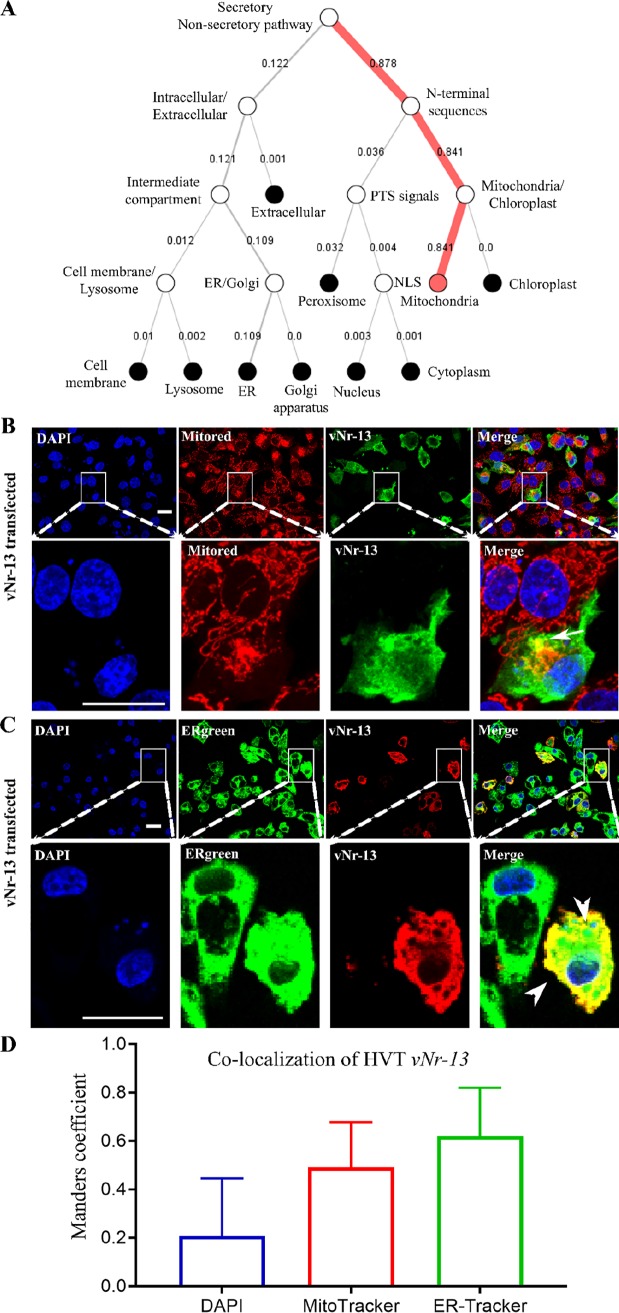
HVT vNr-13 localizes to the mitochondria and endoplasmic reticulum of transfected DF-1 cells. (A) The probability of subcellular localization of HVT vNr-13 was predicted by the DeepLoc-1.0 online algorithm. The hierarchical tree suggests that vNr-13 is localized predominantly to the mitochondrial membrane (0.84) and in a small portion to the ER membrane (0.10). According to immunofluorescence staining, HVT vNr-13 localizes with the mitochondrial network and ER of transfected DF-1 cells. (B and C) The yellow color indicates HVT vNr-13 colocalization with the mitochondrial network (white arrow) and ER (white arrowhead). The second-row images correspond to a higher magnification of the area within the white squares in the first-row images. HVT vNr-13 was stained with monoclonal antibody F878 EG2 (green for mitochondria and red for ER), mitochondria were stained with MitoTracker dye (red), the ER was stained with ER-Tracker dye (green), and nuclei were stained with DAPI (blue). All confocal image scale bars represent 20 μm. (D) The HVT vNr-13 Manders overlap coefficients with nuclei, mitochondria, and ER were 0.21 ± 0.24, 0.49 ± 0.19, and 0.62 ± 0.20, respectively. The data are shown as mean ± standard deviation (SD).

Expression of HVT *vNr-13* in DF-1 cells was examined by immunofluorescence (IF) and Western blot analysis. Figure 2A shows a representative image of IF detection of vNr-13 using the specific F878 EG2 antibody in DF-1 cells transfected with the pcDNA3.1-*vNr-13* plasmid. HVT vNr-13-transfected cells appeared to show diffuse staining throughout the cytoplasm of most cells (Fig. 2A) and a relatively faint nuclear staining with apparent labeling of the nuclear envelope (white arrows). A cyan blue color shows vNr-13 colocalization (yellow arrows) with the nuclear envelope or membrane of the nuclei with a Manders overlap coefficient of 0.21 ± 0.24 (Fig. 2A and 3D). Furthermore, some of the cells appeared rounded, with vNr-13 distributed throughout the cytoplasm and faint nuclear staining with no apparent nuclear envelope labeling. In the past, it was reported that the localization of Bcl-2 orthologs to the nucleus was strongly dependent on the cell cycle and that there was strong expression in the mitotic cells (28). A Western blot image demonstrating the vNr-13 protein as a single band of approximately 19 kDa detected by the F878 EG2 antibody is shown in Fig. 2B. Moreover, HVT vNr-13 was substantially distributed both in the cytosolic and nuclear fractions of transfected cells, as confirmed by Western blotting (Fig. 2C). The purity of subcellular fractions was assayed with anti-α-tubulin or anti-histone H3 antibodies for the cytosolic and nuclear fractions, respectively. A trace amount of α-tubulin was detected in nuclear fraction and of histone H3 in cytosolic fraction, which may be the consequence of incomplete separation of cytosolic and nuclear fractions or the presence of soluble α-tubulin in the nuclear fraction and of soluble histone H3 in cytosolic fraction of the mitotic phases of cells.

### HVT vNr-13 localizes to the mitochondria and ER.

Several Bcl-2 family proteins and Nr-13 orthologs localize to the mitochondria (4, 7, 29). Interestingly, Aouacheria et al. (8) have shown that HVT vNr-13 partly colocalizes with the mitochondria in transfected CEFs, but there is still little information on vNr-13 distribution in other subcellular organelles. Thus, the HVT vNr-13 subcellular localization was investigated by bioinformatics prediction and immunofluorescence analyses. The subcellular localization of the HVT vNr-13 protein was predicted by using the online neural network algorithm DeepLoc-1.0 server (30). A hierarchical tree with multiple nodes and an attention plot (alpha) was developed to understand HVT vNr-13 protein subcellular localization and sorting pathways. The probability score of HVT vNr-13 was higher for mitochondria (0.81) and endoplasmic reticulum (ER) (0.14) than for other subcellular organelles (see Fig. S1 in the supplemental material). The hierarchical tree data suggested that the majority of vNr-13 was localized to the mitochondrial membrane (0.84) and a small portion to ER membrane (0.10) (Fig. 3A and S1). According to attention plot (alpha), a specific interspersed region of HVT vNr-13 between amino acids 150 and 179 was involved in mitochondrial membrane localization (Fig. S1, attention plot). Based on immunofluorescence assay, the HVT vNr-13 protein was significantly colocalized with the mitochondria and ER of transfected DF-1 cells, with Manders overlap coefficients of 0.49 ± 0.19 and 0.62 ± 0.20, respectively (Fig. 3B to D). In Fig. 3B and C, vNr-13 colocalization with the mitochondrial network (white arrow) and ER (white arrowhead) is observed as a yellow color. Thus, the bioinformatics prediction of localization was consistent with the immunofluorescence results.

### CRISPR/Cas9-mediated ablation of the *vNr-13* gene in HVT FC126.

**FIG 4:**
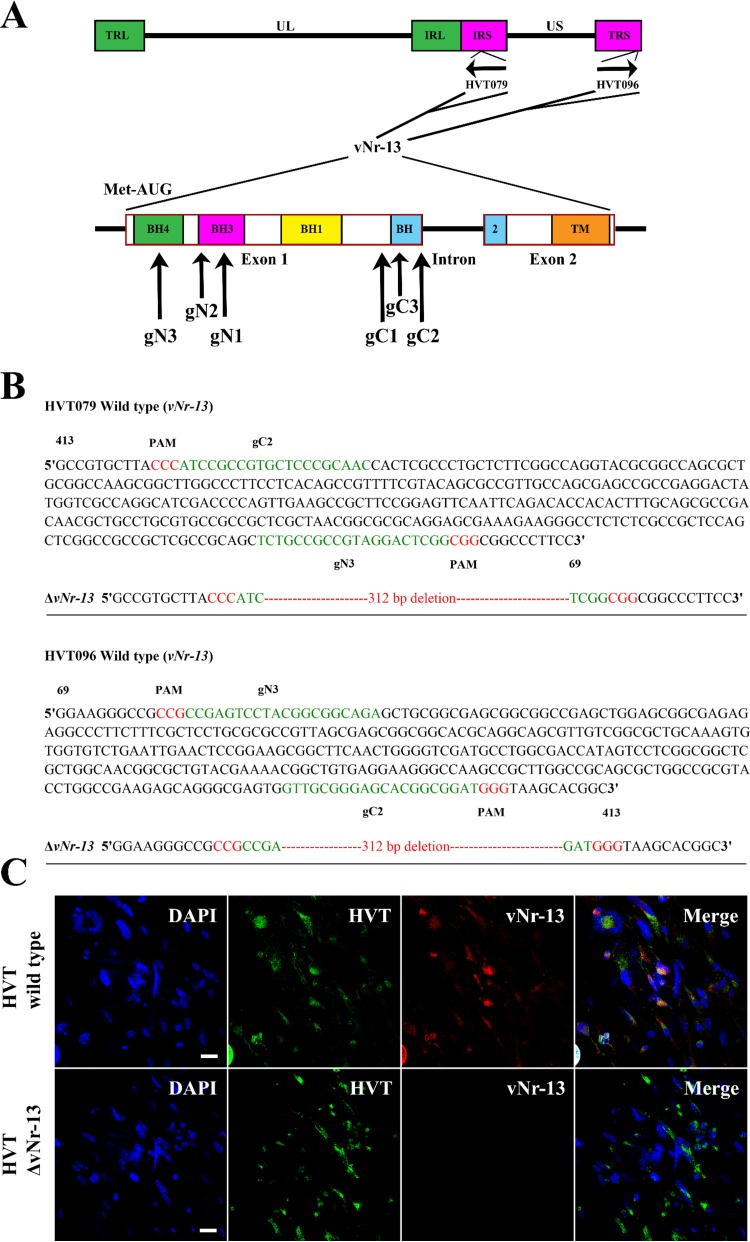
Efficient deletion of HVT *vNr-13* by CRISPR/Cas9 technology. (A) Exon 1 of both HVT079 and HVT096 was targeted by three unique guide RNAs at the 5′ (gN1, gN2, and gN3) and 3′ (gC1, gC2, and gC3) ends in nine combinations, and gN3gC2 was found to be the best combination for efficient deletion. (B) Confirmation of deletion of 312 bp (highlighted in red) in exon 1 of both HVT079 and HVT096 at the gN3 and gC2 target sites by PCR sequencing. In gN3 and gC2, PAM sequences are highlighted in red and guide RNA sequences in green. (C) Confirmation of deletion of HVT *vNr-13* sequences by immunofluorescence staining with anti-vNr-13 monoclonal antibody F878 EG2 (red) and anti-HVT polyclonal serum (green) as an infection control. Scale bar, 25 μm.

To evaluate the function of the *vNr-13* gene in the HVT genome, we chose to knock out both HVT079 and HVT096 using CRISPR/Cas9-based gene editing tools described previously in work from our laboratory (19). First, three unique guide RNA (gRNA) sequences targeting the 5′ (gN1, gN2, and gN3) and 3′ (gC1, gC2, and gC3) regions of exon 1 of *vNr-13* were designed (Table 1 and Fig. 4A) and cloned into pX330A-1x2, which expresses Cas9. To select the most efficient gene editing combination of guide RNAs, three 5′ and three 3′ *vNr-13* exon 1-targeting gRNA/Cas9 constructs were transfected into CEFs in all nine possible combinations (gN1gC1, gN1gC2, gN1gC3, gN2gC1, gN2gC2, gN2gC3, gN3gC1, gN3gC2, and gN3gC3). At 12 h posttransfection, CEFs were infected with the FC126 strain of HVT. The infected CEFs were harvested at 72 h postinoculation (hpi). Mutations in the HVT genome were detected only with the gN3gC2 combination (highlighted in green in Fig. 4B). Plaque purifications of the mutant viruses were performed until the clones were found to contain only pure populations of the expected *vNr-13* exon 1 deletions in both the HVT079 and HVT096 genes. Precise editing of the locus was confirmed by sequence analysis of the PCR product amplified using primers outside the target of HVT079 and HVT096 (Fig. 4B). Furthermore, deletion of *vNr-13* in the mutant virus stocks was confirmed by IF staining with vNr-13-specific F878 EG2 monoclonal antibodies and HVT-specific chicken polyclonal serum. In wild-type HVT-infected cells, both F878 EG2-Alexa 568 and HVT-Alexa 488 staining was detected, whereas in HVT-Δ*vNr-13*-infected cells, only HVT-Alexa 488 was detected. Negative staining with F878 EG2-Alexa 568 demonstrated the lack of expression and successful deletion of *vNr-13* (Fig. 4C).

**TABLE 1:** Primers used for *vNr-13* expression construct cloning, gRNA cloning, and recombinant virus characterization

Category and primer	Sequence (5′ → 3′)
*vNr-13* expression construct primers	
*vNr-13*-expression construct-F	ATTAGGATCCATGGCTGACTCCCTGAAGG
*vNr-13*-expression construct-R	ATTAGAATTCTTAGGATTGGAATTGCCAC
Guide RNAs on 5′ *vNr-13* exon 1	
*vNr-13*-exon1-gN1-F	CACCGGCGTGCCGCCGCTCGCTAA
*vNr-13*-exon1-gN1-R	AAACTTAGCGAGCGGCGGCACGCC
*vNr-13*-exon1-gN2-F	CACCGCCTGCGCGCCGTTAGCGAG
*vNr-13*-exon1-gN2-R	AAACCTCGCTAACGGCGCGCAGGC
*vNr-13*-exon1-gN3-F	CACCGCTGCCGCCGTAGGACTCGG
*vNr-13*-exon1-gN3-R	AAACCCGAGTCCTACGGCGGCAGC
Guide RNAs on 3′ *vNr-13* exon 1	
*vNr-13*-exon1-gC1-F	CACCGCTCGCCCTGCTCTTCGGCC
*vNr-13*-exon1-gC1-R	AAACGGCCGAAGAGCAGGGCGAGC
*vNr-13*-exon1-gC2-F	CACCGTTGCGGGAGCACGGCGGAT
*vNr-13*-exon1-gC2-R	AAACATCCGCCGTGCTCCCGCAAC
*vNr-13*-exon1-gC3-F	CACCGGCGTACCTGGCCGAAGAGC
*vNr-13*-exon1-gC3-R	AAACGCTCTTCGGCCAGGTACGCC
Outside primers	
HVT079-outside-F	AGCAAGACAGCGTCCGATAC
HVT079-outside-R	TTGTGGTGGCGGAACTAATC
HVT096-outside-F	GTGGCGGAACTAATCGGGCG
HVT096-outside-R	ACTCGAGCAAGACAGCGTCC

### Deletion of *vNr-13* affects early *in vitro* replication of HVT in cell culture.

**FIG 5:**
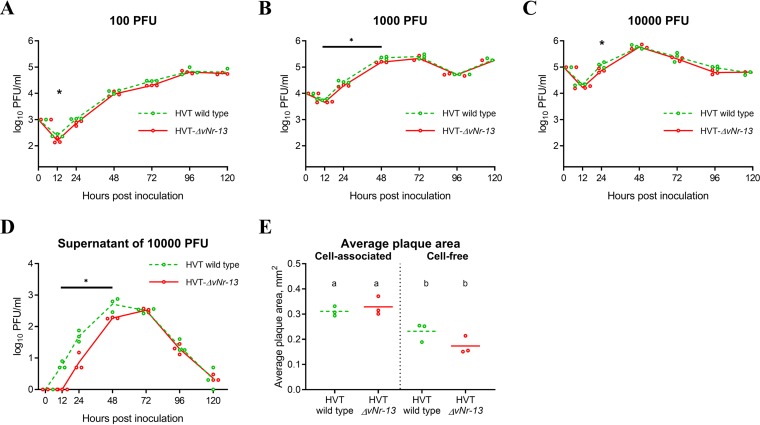
*In vitro* growth kinetics of wild-type HVT and HVT-Δ*vNr-13* on chicken embryo fibroblasts (CEFs). In each experiment, CEF monolayers seeded at 1.3 × 10^6^ were inoculated with wild-type HVT and HVT-Δ*vNr-13*, and viral titers were determined at 0, 12, 24, 48, 72, 96 and 120 h postinoculation (hpi). Growth curves were determined with 100, 1,000, and 10,000 PFU per well, which correspond to multiplicities of infection of 0.00007, 0.0007, and 0.007, respectively. Growth kinetics (means ± SD) were evaluated with three biologically independent experiments. (A) Replication of HVT-Δ*vNr-13* was significantly lower than that of wild-type HVT at 12 hpi in cells inoculated with 100 PFU. (B) In cells inoculated with 1,000 PFU, HVT-Δ*vNr-13* replication was significantly lower than that of wild-type HVT at 12, 24, and 48 hpi. (C) HVT-Δ*vNr-13* showed significantly lower replication than wild-type HVT at 24 hpi in cells inoculated 10,000 PFU. (D) Viral titers were determined in supernatant (cell-free virus) of the samples inoculated with 10,000 PFU to understand the cell-free viral kinetics of wild-type HVT and HVT-Δ*vNr-13*. Cell-free HVT-Δ*vNr-13* showed significantly reduced viral titers compared to those of cell-free wild-type HVT at 12, 24, and 48 hpi. The green dotted line represents the mean values of the wild-type HVT, and the red line represents the mean values of HVT-Δ*vNr-13*. An asterisk indicates a significant difference of viral titers between wild-type HVT and HVT-Δ*vNr-13* (*P* < 0.05). (F) The average plaque areas were determined for both cell-associated and cell-free wild-type HVT and HVT-Δ*vNr-13*. The plaque areas of cell-associated wild-type HVT and HVT-Δ*vNr-13* were significantly higher than those of the cell free virus. Groups with different letters are significantly different from each other (*P* < 0.05).

To determine if the deletion of both copies of *vNr-13* affected the growth kinetics of HVT, we determined the plaque counts and the average plaque areas of wild-type HVT and HVT-Δ*vNr-13* in CEFs. Plaque assay was performed to determine the number of PFU formed at 0, 12, 24, 48, 72, 96, and 120 hpi by titration of wild-type HVT and HVT-Δ*vNr-13* at 100, 1,000, and 10,000 PFU per well, which correspond to multiplicities of infection (MOIs) of 0.00007, 0.0007, and 0.007, respectively. In wells inoculated with 100 PFU, replication of HVT-Δ*vNr-13* was significantly (1.6-fold) lower than that of wild-type HVT at 12 hpi (Fig. 5A). HVT-Δ*vNr-13* replication was significantly lower than that of wild-type HVT at 12, 24 and 48 hpi (by 1.3-, 1.4-, and 1.5-fold, respectively) in wells inoculated with 1,000 PFU (Fig. 5B). Similarly, in wells inoculated with 10,000 PFU, HVT-Δ*vNr-13* produced significantly (1.7-fold) lower replication at 24 hpi than did wild-type HVT (Fig. 5C). Furthermore, the highest titers produced with both wild-type HVT and HVT-Δ*vNr-13* were only 5.7 × 10^5^ PFU/ml, even with the highest, 10,000-PFU inoculation (10^5^ PFU/ml). The overall low titers in the range of 10^5^ PFU/ml are mainly due to the strictly cell-associated nature of HVT in *in vitro* cell culture. Thus, the 1.3- to 1.7-fold-lower levels of cell-associated virus in HVT-Δ*vNr-13* compared to wild-type HVT with different PFU cannot be underestimated.

The above results also prompted us to determine the growth kinetics of cell-free virus in the culture supernatant of the infected cells. Thus, we determined the titers of the virus in the culture supernatants of cells inoculated with 10,000 PFU of the virus. In both wild-type- and HVT-Δ*vNr-13*-infected culture supernatants, virus titers were low, which was consistent with previous studies (22, 31) (Fig. 5D). Moreover, initiation of the replication was delayed in cell-free virus, especially with HVT-Δ*vNr-13* at 12 and 24 hpi, and after 48 hpi retarded replication was observed in both wild-type HVT and HVT-Δ*vNr-13* compared with cell-associated virus. Specifically, at 12 hpi viral plaques were not observed with cell-free HVT-Δ*vNr-13*, whereas only 6 PFU was observed with cell-free wild-type HVT. Cell-free virus of HVT-Δ*vNr-13* showed significantly reduced viral titers compared to cell-free virus of wild-type HVT at 24 and 48 hpi (by 6.2- and 3-fold, respectively). Taken together, cell-free viral growth kinetics of wild-type HVT and HVT-Δ*vNr-13* substantially corroborated the above-observed kinetics of the cell-associated virus at early time points. Determination of the growth kinetics from supernatants samples inoculated with low PFU (100 and 1,000 PFU) was not considered because of above observed limited replication from high-PFU-inoculated wells. Furthermore, the average plaque areas were measured with both cell-associated and cell-free virus-infected plaques of wild-type HVT and HVT-Δ*vNr-13* (Fig. 5E). Determination of average plaque areas demonstrated that there was no significant difference between wild-type HVT and HVT-Δ*vNr-13* plaque areas.

### HVT *vNr-13* expression leads to disruption of mitochondrial morphology.

**FIG 6:**
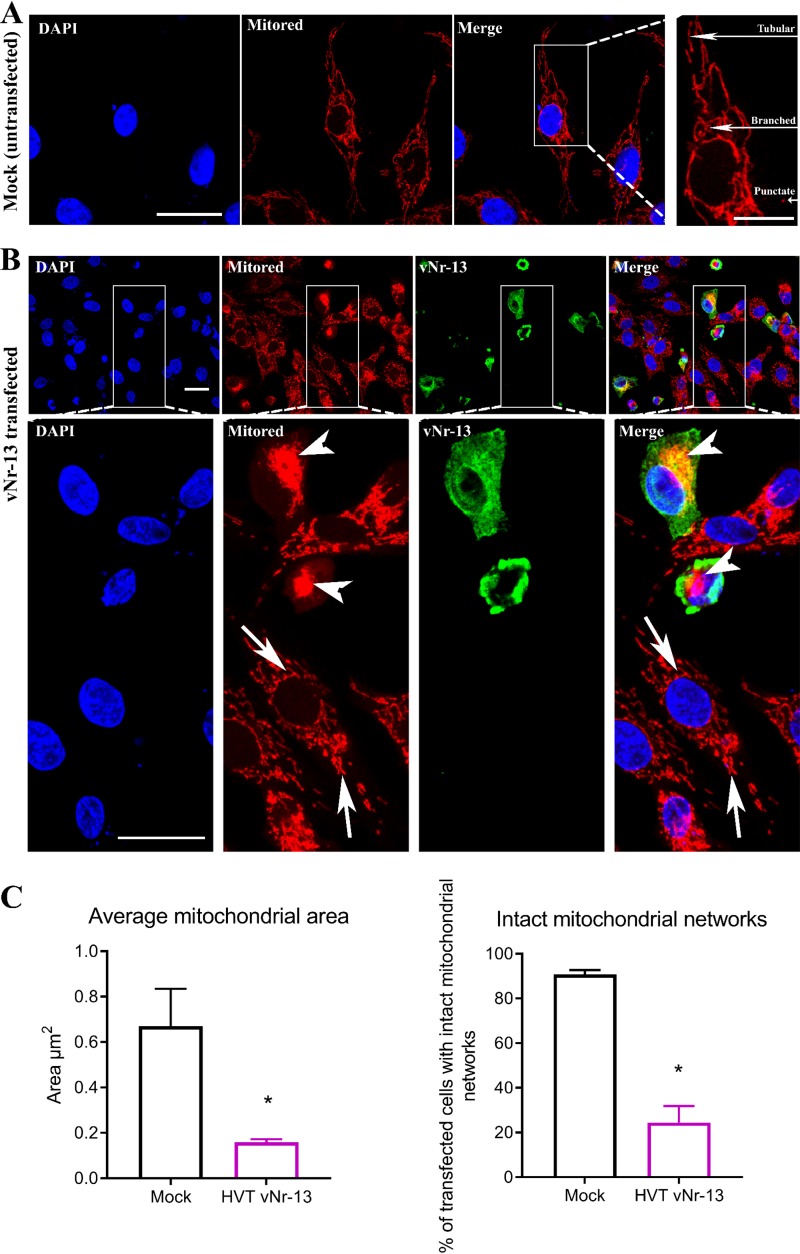
HVT vNr-13 disrupts the mitochondrial network morphology in transfected DF-1 cells. (A) In the cytoplasm of untransfected cells, clearly dispersed staining of tubular, branched, and punctate structures was observed. Scale bar, 25 μm. The right panel shows a higher-magnification image with clear tubular, branched, and punctate structures labeled (scale bar, 10 μm). (B) After HVT vNr-13 transfection, changes in mitochondrial morphology were observed by confocal microscopy. A yellow color indicates HVT vNr-13 colocalization with the disrupted mitochondrial network (arrowhead) in vNr-13-transfected DF-1 cells. The second-row images correspond to a higher magnification of the area within the white squares in the first-row images. Clear tubular, branched, and punctate mitochondrial structures were observed in nontransfected cells (arrows). HVT vNr-13 was stained with monoclonal antibody F878 EG2 (green), and mitochondria were stained with MitoTracker dye (red). Scale bar, 25 μm. (C) The average mitochondrial area and intact mitochondrial networks in a cell were determined as described in Materials and Methods. The average mitochondrial area and intact mitochondrial networks were significantly lower in HVT vNr-13-transfected cells than in nontransfected cells. Data are shown as means from three biologically independent experiments ± standard deviation (SD). An asterisk indicates a significant difference (*P* < 0.05).

Many studies have reported that alphaherpesviruses such as pseudorabies virus (PRV), human herpes simplex virus 1 (HSV-1), and HSV-2 play important roles in the mitochondrial distribution and morphology in infected cells (14, 16, 18, 32). However, no information is available on the role of HVT vNr-13 in modulating the morphology of the mitochondrial networks. We investigated changes in mitochondrial morphology in HVT vNr-13-transfected cells by confocal microscopy. In mock-transfected cells, the mitochondrial network was seen evenly distributed throughout the cell and was characterized by the normal three shapes of the tubular, branched, and punctate mitochondrial network (Fig. 6A) (16). In HVT vNr-13-transfected cells, the majority of the vNr-13 protein-colocalized mitochondrial structures were abnormally aggregated around the nuclear periphery (Fig. 6B), and some were irregularly distributed in the cytoplasm. The average mitochondrial area in mock-transfected cells was 0.7 ± 0.2 μm, whereas the mitochondrial area was only 0.2 ± 0.0 μm in vNr-13-transfected cells. Furthermore, while approximately 90.7% ± 2.1% of cells possessed intact mitochondrial networks in the mock-transfected cells, only 24.5% ± 7.4% of vNr-13-transfected cells retained intact mitochondrial networks (Fig. 6C).

### Disrupted mitochondrial morphology is substantially reduced in HVT-Δ*vNr-13* infection.

**FIG 7:**
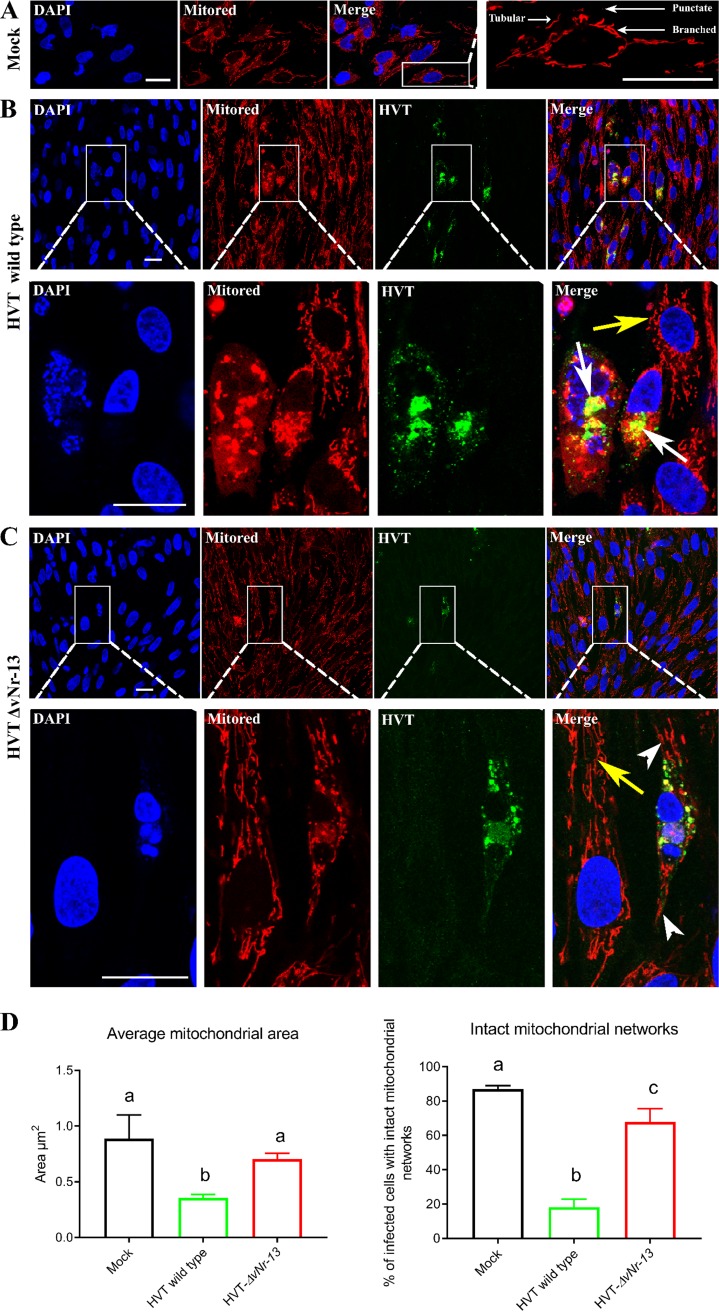
The mitochondrial network morphology is disrupted during wild-type HVT infection in CEFs. (A) Clearly dispersed tubular, branched, and punctate mitochondrial structures were observed in mock-infected CEFs. (B) After wild-type HVT infection, the mitochondrial morphology was severely disrupted and aggregated near the nucleus (white arrows). A yellow color indicates vNr-13 colocalization with the mitochondrial network. The second-row images correspond to a higher magnification of the area within the white squares in the first-row images to show severe disruption of mitochondrial network morphology (white arrows). (C) HVT-Δ*vNr-13* infection caused lower mitochondrial network disruption than wild-type HVT infection (arrowhead). The second-row images correspond to a higher magnification of the area within the white squares in the first-row images to show the better intact mitochondrial network morphology than with wild-type HVT (arrowhead). A clearly dispersed intact mitochondrial structure was observed in noninfected cells from both wild-type HVT and HVT-Δ*vNr-13* conditions (yellow arrow). Chicken polyclonal anti-HVT antibodies were used to label HVT antigens (green), and mitochondria were stained with MitoTracker dye (red). All confocal image scale bars represents 25 μm. (D) The average mitochondrial area and intact mitochondrial networks in a cell were determined as described in Materials and Methods. The average mitochondrial area and intact mitochondrial networks were significantly lower in the wild-type-HVT-infected cells than in the HVT-Δ*vNr-13*-infected and mock-infected cells. Data are shown as means from three biologically independent experiments ± standard deviation (SD). Bars with different letters are significantly different from each other (*P* < 0.05).

Having demonstrated that *vNr-13* expression leads to the disruption of mitochondrial networks, we wanted to examine the effect of infection with wild-type HVT or *vNr-13* deletion mutant viruses on the morphology of the mitochondrial networks. In the uninfected CEFs, an evenly spread tubular, branched, and punctate mitochondrial network was observed (Fig. 7A). Mitochondrial network dynamics were severely disrupted following wild-type HVT infection, which appeared to colocalize with mitochondria and substantially aggregate near the nucleus (Fig. 7B), while HVT-Δ*vNr-13* infection caused much-reduced disruption of the mitochondrial network and the colocalized mitochondrial structures were irregularly distributed in the cytoplasm in comparison with wild-type HVT infection (Fig. 7C). The average mitochondrial area of mock-infected cells was 0.9 ± 0.1 μm, while it was only 0.4 ± 0.0 μm wild-type-HVT infected cells. In HVT-Δ*vNr-13*-infected cells, the average mitochondrial area was 0.7 ± 0.0 μm, which was significantly higher than that for wild-type HVT. In addition, approximately 87.0% ± 1.9% of mock-infected cells possessed intact mitochondrial networks, whereas only 18.2% ± 4.8% of wild-type-HVT infected cells retained intact mitochondrial networks (Fig. 7D). In contrast, 67.9% ± 7.7% of HVT-Δ*vNr-13*-infected cells possessed intact mitochondrial networks, which was significantly higher than the value for wild-type-HVT-infected cells and significantly lower than that for mock-infected cells. These data indicated that vNr-13 protein was involved in the disruption of mitochondrial network morphology in wild-type-HVT-infected cells, which was substantially restored in HVT-Δ*vNr-13*-infected cells.

### HVT vNr-13 inhibits apoptosis compared to the strong apoptosis inducer apoptin.

**FIG 8:**
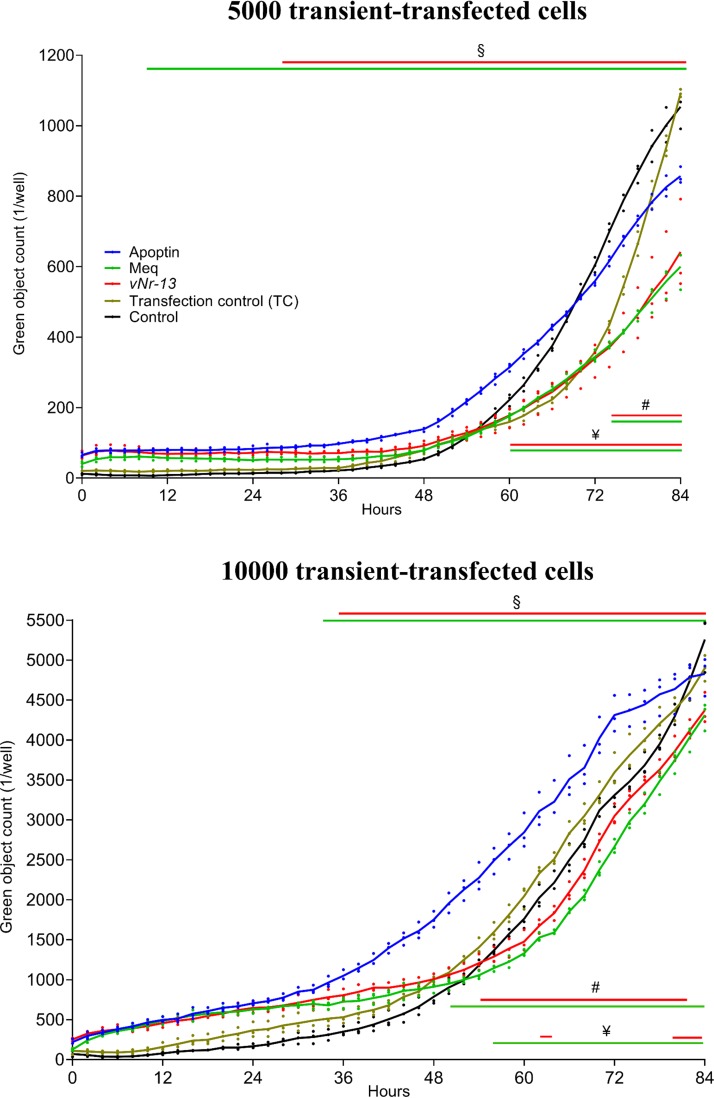
HVT vNr-13 inhibits apoptosis compared to the strong apoptosis inducer apoptin. Apoptotic cell kinetics of HVT vNr-13-transfected cells were compared with those of apoptin- and Meq-transfected cells as inducer and inhibitor controls of apoptosis, respectively. Apoptosis kinetics were determined by noninvasive high-throughput IncuCyte S3 real-time monitoring. Fluorescent images from the IncuCyte S3 live-cell imaging system were collected with 5,000 and 10,000 transiently transfected cells at 2-h intervals for 84 h. HVT vNr-13-transfected cell apoptosis was significantly lower than that of apoptin-transfected cells between 28 and 84 h with 5,000 transiently transfected cells and between 36 and 84 h with 10,000 transiently transfected cells. In the Meq-transfected cells, the level of apoptosis was significantly lower than that in the apoptin-transfected cells between 10 and 84 h with 5,000 cells and between 34 and 84 h with 10,000 cells. The apoptosis kinetics of vNr-13- and Meq-transfected cells were significantly lower than those of transfection control cells between 74 and 84 h and of control cells between 60 and 84 h with 5,000 cells, while with 10,000 cells, the apoptosis kinetics of vNr-13-transfected cells were significantly lower than those of transfection control cells between 54 and 82 h and of control cells at 62, 82, and 84 h. In addition, with 10,000 cells, the Meq-transfected cell apoptosis kinetics were significantly lower than those of transfection control cells between 50 and 84 h and of control cells between 56 and 84 h. §, #, and ¥ indicate apoptosis kinetics of apoptin-transfected, transfection control, and control cells, respectively, that were significantly higher than those of vNr-13- and Meq-transfected cells (*P* < 0.05). Growth curves of transfected cells are shown as means from three biologically independent experiments ± standard deviation (SD). Transfection and caspase 3/7-positive controls were used.

Following on the previous report demonstrating the inhibition of apoptosis by vNr-13 in CEFs (8), we examined the kinetics of vNr-13-induced apoptosis in DF-1 cells, particularly in comparison with apoptin, a strong inducer of apoptosis, and Meq, an inhibitor of apoptosis (33). Fluorescent images from the IncuCyte S3 live-cell imaging system were collected with 5,000 and 10,000 transiently transfected cells at every 2 h for 84 h (Fig. 8). Kinetics of apoptosis quantitated from the fluorescent images showed that apoptosis was significantly lower in the vNr-13-transfected cells than in the apoptin-transfected cells, between 28 and 84 h with 5,000 transiently transfected cells and between 36 and 84 h with 10,000 transiently transfected cells. In the Meq-transfected cells, the level of apoptosis was significantly lower than that in the apoptin-transfected cells between 10 and 84 h with 5,000 cells and between 34 and 84 h with 10,000 cells. The apoptosis kinetics of transfection control cells were significantly lower than those of vNr-13-, apoptin-, and Meq-transfected cells, between 0 and 40 h with 5,000 cells and between 0 and 30 h with 10,000 cells. In control cells, the apoptosis kinetics were significantly lower than with vNr-13-, apoptin-, and Meq-transfected cells, between 0 and 50 h with 5,000 cells and between 0 and 46 h with 10,000 cells. The apoptosis kinetics of vNr-13- and Meq-transfected cells were significantly lower than those of transfection control cells between 74 and 84 h and of control cells between 60 and 84 h with 5,000 cells, while with 10,000 cells, the apoptosis kinetics of *vNr-13*-transfected cells were significantly lower than those of transfection control cells between 54 and 82 h and of control cells at 62, 82, and 84 h. Furthermore, with 10,000 cells, the Meq-transfected cells apoptosis kinetics were significantly lower than those of the transfection control between 50 and 84 h and of the control between 56 and 84 h. Our data also showed that Meq was a strong inhibitor of apoptosis, as has been demonstrated previously (33), in comparison to vNr-13. Thus, our study has demonstrated that IncuCyte S3-based real-time monitoring is a very valuable method to examine the vNr-13 apoptosis kinetics with apoptin- and Meq-transfected cells as an inducer and an inhibitor of apoptosis, respectively.

### HVT vNr-13 inhibition of apoptosis varies with virus titer and serum deprivation.

**FIG 9:**
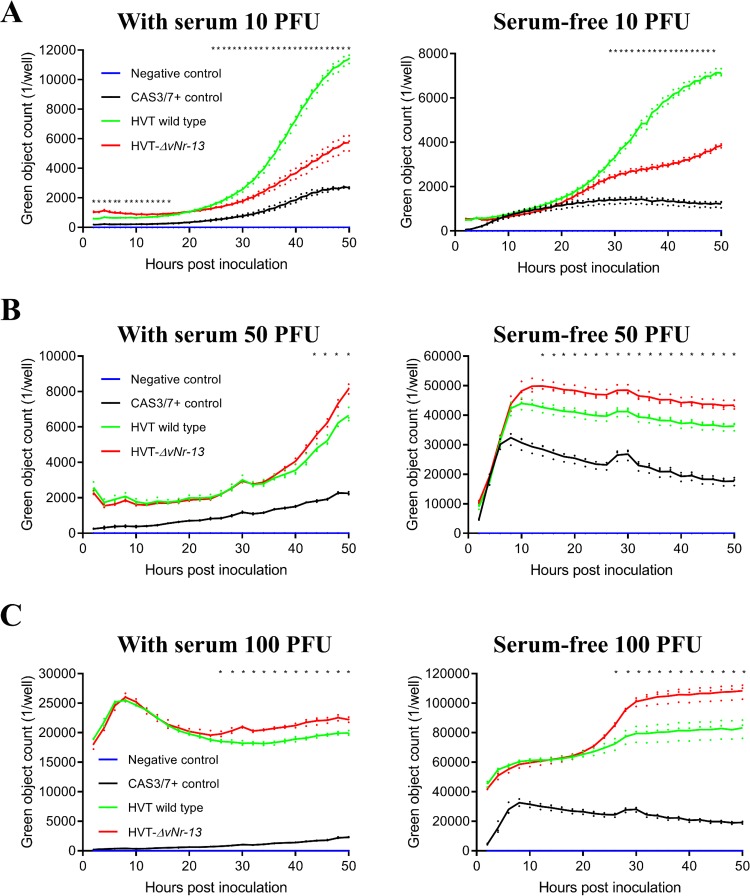
HVT vNr-13 inhibition of apoptosis varies with virus titer and serum deprivation. The apoptotic cell kinetics for wild-type HVT and HVT-Δ*vNr-13* were monitored with serum or under serum-free conditions with the high-throughput IncuCyte S3 real-time system. CEFs (4.5 × 10^4^ cells per well) in a 96-well plate were inoculated with wild-type HVT and HVT-Δ*vNr-13* at 10, 50, and 100 PFU per well, which correspond to MOIs of 0.0002, 0.001, and 0.02, respectively, and then caspase 3/7 reagent was added to the cells. Real-time IncuCyte fluorescent images were captured every 1 h for wells inoculated with 10 PFU and every 2 h for wells inoculated with 50 and 100 PFU for 50 h. (A) Quantitation of green fluorescent images as an apoptosis indicator showed that apoptosis was significantly higher between 2 and 16 hpi in the HVT-Δ*vNr-13*-infected cells than in the cells infected with wild-type HVT at 10 PFU with serum, and no difference was observed under serum-free conditions. Further, after 25 hpi with serum and 28 hpi without serum, the apoptotic cells were significantly higher in the wild-type-HVT-infected cells than in the HVT-Δ*vNr-13*-infected cells when 10 PFU was used. (B) In wells inoculated with 50 PFU, the apoptosis was significantly higher in the HVT-Δ*vNr-13* infected cells than in the wild-type-HVT-infected cells between 44 and 50 hpi with serum and between 14 and 50 hpi under serum-free conditions. (C) In wells inoculated with 100 PFU, the apoptosis was significantly higher between 26 and 50 hpi in the HVT-Δ*vNr-13* infected cells than in the cells infected with the wild-type HVT both with serum and under serum-free conditions. Mean values for the wild-type HVT, HVT-Δ*vNr-13*, caspase 3/7 control, and negative control are represented as green, red, black, and blue lines, respectively. Apoptotic cell kinetics of infected cells are shown as means from nine biological independent experiments ± standard deviation (SD). An asterisk indicates a significant difference (*P* < 0.05).

As the Bcl-2 homologs are known for apoptosis inhibition, we compared the levels and kinetics of apoptosis between wild-type HVT and HVT-Δ*vNr-13* in serum and serum-free medium using the caspase 3/7 apoptosis assay reagent. The IncuCyte S3 live-cell imaging system was used for monitoring of apoptosis on CEFs infected with wild-type HVT and HVT-Δ*vNr-13* at 10, 50, and 100 PFU per well, which corresponds to MOIs of 0.0002, 0.001, and 0.02, respectively (Fig. 9). Quantitation of apoptosis was carried out for 50 h from the fluorescent images collected at every 1 h for wells inoculated with 10 PFU and every 2 h for wells inoculated with 50 and 100 PFU. In wells inoculated with 10 PFU, the levels of apoptosis were significantly higher between 2 and 16 hpi in the HVT-Δ*vNr-13*-infected cells than in the cells infected with the wild-type HVT in the presence of serum. However, under serum-free conditions, no difference in the apoptosis kinetics was observed between 2 and 16 hpi in the wild-type-HVT- and HVT-Δ*vNr-13*-infected cells. However, from 25 hpi with serum and 28 hpi with serum-free medium, the levels of apoptosis were significantly higher in the wild-type-HVT-infected cells than in HVT-Δ*vNr-13*-infected cells at 10 PFU (Fig. 9A). In wells inoculated with 50 PFU, the apoptosis was significantly higher in the HVT-Δ*vNr-13*-infected cells than in the cells infected with the wild-type HVT between 44 and 50 hpi in the presence of serum and between 14 and 50 hpi under serum deprivation conditions (Fig. 9B), while in wells inoculated with 100 PFU, the apoptosis was significantly higher between 26 and 50 hpi in the HVT-Δ*vNr-13*-infected cells than in the cells infected with the wild-type HVT under conditions of both serum presence and deprivation (Fig. 9C). To simplify the explanation of the apoptosis kinetics results from the IncuCyte S3 data, we will here refer to 10 PFU as low PFU and to 50 and 100 PFU as high PFU.

Taking the results together, with low-PFU infection in the presence of serum, the apoptosis kinetics of HVT-Δ*vNr-13* were higher than those of the wild type at early time points, but no difference was observed under serum-free conditions. However, the apoptosis kinetics of wild-type HVT were higher than those of HVT-Δ*vNr-13* at later time points for low-PFU infection both in the presence of serum and under serum-free conditions. With high PFU, the apoptosis kinetics of HVT-Δ*vNr-13* were higher than those of wild-type HVT at later time points under both serum and serum-free conditions, but no difference was observed at early time points, except for 50 PFU under serum-free conditions. Therefore, vNr-13-based clear inhibition of apoptosis was associated with higher-PFU infection under both serum and serum-free conditions, especially at later time points. Apoptosis kinetics were evaluated in nine biological independent experiments (mean ± standard deviation [SD] is reported).

### Noninfected bystander cells undergo apoptosis.

**FIG 10:**
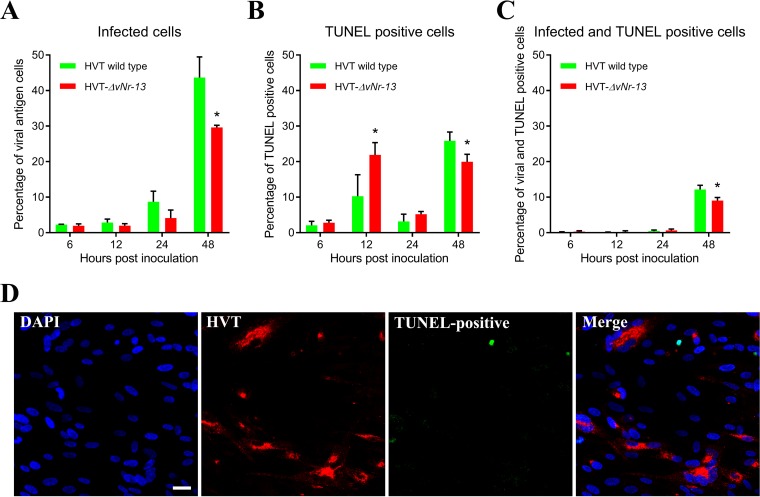
Replication kinetics and viability of wild-type-HVT- and HVT-Δ*vNr-13*-infected cells determined by TUNEL assay. (A) Monolayers of CEFs were inoculated with wild-type HVT and HVT-Δ*vNr-13* at 10 PFU per well, which corresponds to an MOI of 0.00007. After virus inoculation, large number of cells were infected. Wild-type-HVT-infected cells were significantly higher than HVT-Δ*vNr-13*-infected cells at 48 hpi. (B) TUNEL-positive cells were observed for both wild-type HVT and HVT-Δ*vNr-13*. TUNEL-positive cells with HVT-Δ*vNr-13* were significantly higher at 12 hpi than those with wild-type HVT. (C) At 48 hpi, TUNEL-positive and infected TUNEL-positive cells were lower for HVT-Δ*vNr-13* than for wild-type HVT. An asterisk indicates a significant difference (*P* < 0.05). (D) A representative confocal image illustrating TUNEL-positive cells (apoptotic; green) in the vicinity of HVT-infected cells (red) but not in the HVT-infected cells. HVT-infected cells were in general not apoptotic. Scale bar, 25 μm.

Inoculation of wild-type HVT and HVT-Δ*vNr-13* virus at 10 PFU per well, which corresponds to an MOI of 0.00007, on CEFs resulted in the infection of large numbers of cells. Based on the quantitation of virus-infected cells, wild-type HVT-infected cells showed marginal increases in infection at 6, 12, and 24 hpi, but these levels were significantly higher than those for HVT-Δ*vNr-13* at 48 hpi (Fig. 10A). We also quantified the levels of apoptosis by terminal deoxynucleotidyltransferase-mediated dUTP-biotin nick end labeling (TUNEL) assay in CEFs infected by the two viruses. While TUNEL-positive cells were detected in cells infected by both viruses, the numbers were significantly higher in the HVT-Δ*vNr-13*-infected cells than in wild-type-HVT-infected cells, especially at 12 hpi (Fig. 10B). Interestingly, the TUNEL-positive cells appeared to be mainly noninfected cells present in the vicinity of infected cells, many of which were TUNEL negative (Fig. 10C and D), suggesting that HVT can induce apoptosis in adjacent noninfected cells. A representative confocal image illustrating TUNEL-positive cells in the vicinity of HVT-infected cells but not in the HVT-infected cells is shown in Fig. 10D. At 48 hpi, TUNEL-positive and infected TUNEL-positive cells were lower for HVT-Δ*vNr-13* than for the wild-type HVT.

## DISCUSSION

Many viruses encode vBcl-2 proteins to evade or modulate the apoptosis mechanisms of the host (6, 7, 34–36). In gammaherpesviruses, vBcl-2 proteins play an important role in the inhibition of apoptosis and dissemination of virus (6, 7, 34, 35). Although the HVT genome organization shows extensive similarity with that of the MDV genome, HVT is the only avian alphaherpesvirus which encodes a Bcl-2 homolog, vNr-13, expressed from two identical copies from the genes HVT079 and HVT096 (9, 23). Several MDV and infectious laryngotracheitis virus (ILTV) proteins were shown to have antiapoptotic properties, but none of them have Bcl-2 homology (33, 37, 38). Multiple-sequence alignments revealed that vNr-13 shared the highest sequence identity of 64.4% with chicken Nr-13, compared to only 30% with the frog homolog (10, 11). Similarly, Nrh, a human homolog of Nr-13 protein, and Boo/Diva Bcl-2 proteins of mouse and rat also shared relatively low identity (30% and 26%, respectively) with vNr-13 (10). Moreover, the organization of the HVT *vNr-13* exon/intron structure and the four BH domains was highly conserved with Nr-13 proteins, strongly supporting the notion that it is a true Nr-13 ortholog (8, 10, 11). Indeed, Aouacheria et al. (8, 10) have reported earlier that Nr-13 orthologs have same organization and are aligned together. Similar to the case for other Nr-13 orthologs, vNr-13 is functionally classified under the typical Bcl-2 group of prosurvival family proteins (5, 10), with a conserved NWGR motif in the BH1 domain and a GGW motif in the BH2 domain, further providing a clear indication that *vNr-13* may have originated from host gene. Indeed, Bcl-2 family proteins are known to be prototypical members that are highly conserved in evolution from flies and nematodes to humans, and viruses should have copied the Bcl-2 region for their advantage (39).

In 3D structural protein models, the C score estimates the quality of predicted models, and a score of between −5 and 2 defines the range of confidence for the predicted model (40). The C score of 0.25 for vNr-13 signifies that it is a higher-value model with a high confidence. The template modeling (TM) score and root mean square deviation (RMSD) give information on structural similarity with other structures and correlation with the C score. The predicted structure of HVT vNr-13 was observed to be similar to those of conserved Bcl-2-like fold proteins, with eight α-helices and a TM domain (41). Moreover, vNr-13 was closer structurally to Nr-13 of zebrafish (6h1nA) and Bax (1f16A) and functionally to Bcl-xL (2yj1C) and Mcl-1 (4wmrA) (5, 42–44). Nr-13 of zebrafish, Bcl-xL, and Mcl-1 are prosurvival proteins, and Bax is proapoptotic protein. Among Nr-13 orthologs, only Nr-13 of the zebrafish structure has been solved to date, and it has been reported to bind Bad and Noxa proapoptotic proteins at the BH3 motif, which is a unique and promiscuous apoptosis inhibition mechanism, unlike that of other Bcl-2 prosurvival proteins (5). HVT vNr-13 may inhibit apoptosis through a mechanism similar to that for the Nr-13 of zebrafish.

Our study has shown that vNr-13 diffusely distributed throughout the cytoplasm of most cells and shows a relatively faint nuclear staining with apparent labeling of the nuclear envelope. Further, vNr-13 was localized in mitochondria and the ER. Indeed, a unique feature of Bcl-2 homologs is to localize within the outer mitochondrial membrane, interact each other, and homo- or heterodimerize with cellular partners to regulate apoptosis (4). For example, human Nrh localizes to the mitochondria and nuclear envelope, and NRZ localizes to the mitochondria to antagonize proapoptotic proteins Bcl-Xs and Bax (10, 29). We do not know whether vNr-13 functions through such a pathway, and further studies are required to examine whether vNr-13 is localized to the outer or inner membrane of the mitochondria and what interaction partners are involved in the mitochondrial network dynamics and/or pathways of apoptosis inhibition. However, in chickens there are limitations regarding the availability of specific reagents, such as antibodies against cellular binding partners and organelles, and further studies would require development of the necessary reagents.

Based on the previous studies showing that vNr-13 protein can inhibit apoptosis induced by serum deprivation as well as reduce cell proliferation (8), we hypothesized that vNr-13 has important functional roles in the context of HVT replication kinetics in avian cells. In order to test this hypothesis, we used a *vNr-13* deletion mutant of HVT to examine its role in the replication cycle, mitochondrial network morphology, and apoptosis kinetics in comparison to the wild-type HVT. Thus, the CRISPR/Cas9 editing method was followed with nine combinations of gRNAs to delete exon 1 of the *vNr-13* gene. The clear ablation of 312 bp of exon 1 of the *vNr-13* gene was observed only with the gN3gC2 gRNA combination. The exact reason why gN3gC2 combination worked was elusive, but it is known that there are several factors play a role in the efficient and precise deletion of a gene by CRISPR/Cas9 approach (45, 46). First, CRISPR/Cas9 system efficacy varies mainly with the gRNA specificity and efficiency at the target site, efficiency of plasmid DNA delivery, cell types, and PFU of virus (45). Second, effective gRNA designs depend on high-quality genome sequence, effectiveness metrics, and gene annotations (46). In addition, judicious selection of gRNAs with minimal off-target effects is crucial to achieve precise and efficient deletion (45, 46). Therefore, these parameters may have been perfectly optimized for the gN3gC2 combination in this study to delete 312 bp of exon 1 of the *vNr-13* gene. Furthermore, after many passages, the deletion remained stable. In the past 5 years, CRISPR/Cas9-based high-throughput gene editing strategies have been emerging at a fast pace to improve efficiency, specificity, and versatility. Recently, large DNA fragments, introns, and long noncoding RNA deletions have been successfully achieved to investigate gene functions and regulatory roles (47, 48). Therefore, more studies should be performed on the regulatory role of introns between exons 1 and 2 and/or association of the of *vNr-13* gene with exons 1 and 2.

Based on the *in vitro* growth kinetics, *vNr-13* deletion caused no drastic impairment of HVT replication, although a consistent 1.3- to 1.7-fold-lower viral yield at early time points was particularly notable for cell-associated HVT-Δ*vNr-13*. Moreover, a 3- to 6.2-fold decrease in the cell-free HVT-Δ*vNr-13* growth at early time points suggested that deletion of *vNr-13* might have affected the initial events in HVT replication. The highly cell-associated nature of HVT is one of the major limitations in understanding the initial cell entry and early replication events. Although cell-free virus stocks can be used to overcome some of the experimental difficulties for such studies, the titers of cell-free wild-type HVT and HVT-Δ*vNr-13* were extremely low, producing significantly smaller plaques and retarded replication after 48 hpi compared to the cell-associated viruses. Moreover, it has previously been demonstrated that high-titer stocks of cell-free HVT generated by continuous cell culture passages were replication defective *in vivo* (31). Thus, the contribution of cell-free HVT to overall viral growth and kinetics of apoptosis after 48 hpi might be negligible.

Using both an *vNr-13*-expressing plasmid and wild-type and deletion mutant HVTs, we demonstrated for the first time that the reduction in area of mitochondria and disturbance in the mitochondrial network morphology were directly associated with *vNr-13* expression. Our studies clearly showed that the HVT-associated changes in mitochondrial network morphology are at least one of the major effects of vNr-13 on the pathophysiological changes observed in the HVT-infected cells. Indeed, the importance of the disruption of mitochondrial morphology and motility for efficient virus growth and spread mediated through viral glycoprotein B has been shown for other pathogenic herpesviruses, such as pseudorabies virus (14).

The mitochondrial network morphology and dynamics are regulated by the process of mitochondrial fission and fusion in the cells. The single mitochondrion undergoes several fission and fusion events in its physiological life cycle, and these are mediated by several respective machinery proteins. The activation of the fission machinery and, eventually, associated neutralization of the fusion machinery control the mitochondrial shape and/or apoptosis (49). The Bcl-2 family proteins are reported to be involved in the maintenance of mitochondrial networks by mechanisms governing fission/fusion events, but there is sparse information on their functions (50). The human cytomegalovirus UL37x1 gene product, viral mitochondrion-localized inhibitor of apoptosis (vMIA), has been shown to regulate mitochondrial fission/fusion dynamics by binding and inhibiting proapoptotic Bcl-2 family members Bax and Bak (17, 51). It is not known whether vNr-13-associated disruption of the mitochondrial morphology is initiated due to an increased rate of fission or decreased rate of fusion, and further studies are needed to explore the molecular events and interactions of vNr-13.

Although speculative, a hypothesis can be formed to explain the inhibition of apoptosis at early time points in the replication cycle with low-PFU infection in serum-containing medium and at late time points with high-PFU infection under both serum and serum-free conditions. With low-PFU infection in the presence of serum, the restricted viral replication might have favored the increase of noninfected apoptotic cells at earlier time points in HVT-Δ*vNr-13* than in wild-type HVT, but serum deprivation completely restored the increased number of apoptotic cells. Although this observation of no difference in the apoptosis kinetics between wild-type and mutant HVT during serum deprivation was surprising, it might be due to a compensatory effect of another antiapoptotic cellular factor(s). In addition, with high-PFU infection, this unknown compensatory cellular mechanism might still be efficient in regulation of events at early time points, where no difference in the kinetics of apoptotic cells between HVT-Δ*vNr-13* and wild-type HVT was observed, whereas at later time points under high-PFU infection, the increased viral replication may have caused the shutoff of the cellular antiapoptotic mechanism, which may have eventually led to a substantial increase of apoptotic cells, particularly under serum-free conditions. Moreover, at later time points with high-PFU infection, wild-type HVT seemed to protect cells from apoptosis compared to the infections with HVT-Δ*vNr-13*, especially under serum-deprived conditions. Thus, the reduction in the number of apoptotic cells in wild-type HVT compared to HVT-Δ*vNr-13* infections both with serum and under serum-free conditions (particularly at later time points in high-PFU infection) demonstrated that *vNr-13* expression is part of the HVT strategy to maintain cell viability for longer periods to help sustain virus production. Indeed, in infections with many other viruses, such as Epstein-Barr virus (EBV), Kaposi sarcoma-associated herpesvirus (KSHV), murine cytomegalovirus (MCMV), human cytomegalovirus (HCMV), myxomavirus, vaccinia virus, and adenoviruses, it was shown that deletion of vBcl-2 homologs triggered apoptosis (52–57). In human immunodeficiency virus (HIV) infection, it was also observed that inhibition of apoptosis was associated with increased viral production and sustained cell viability (36). Furthermore, in the past, several studies have shown that vBcl-2 proteins of gammaherpesviruses such as KSHV, EBV, and murine gamma herpesvirus 68 (MHV-68) have additional roles in viral lytic replication and/or reactivation, latency and persistent infection, virulence, and virion assembly (7, 53, 58, 59). In KSHV, the viral lytic replication was reported not to be associated with the antiapoptotic and antiautophagic functions (7, 58, 59). As HVT is nonpathogenic and nononcogenic, there are no *in vitro* models or latently infected cell lines available to examine the other potential roles of vNr-13 in latency or reactivation. However, future *in vivo* studies in experimentally vaccinated chickens with intact and *vNr-13*-deletion mutants of HVT can help in examining its potential roles in latency and reactivation.

As a successful *in ovo* vaccine against Marek’s disease and a live recombinant viral vector against multiple avian pathogens (60, 61), replication of HVT in the late-stage embryonic tissues is critical for inducing protection (62). Does vNr-13 play a role in supporting virus replication and inhibiting apoptosis in the late stages of developing embryonic tissues? This is an important question that needs to be addressed to gain insights into the immune responses to vaccines as well as the development of the avian immune system itself. Both the chicken Nr-13 and zebrafish NRZ were shown to be expressed at high levels in developmental stages of embryonic bursa and somitogenesis, respectively, where they had functional roles in the inhibition of apoptosis during crucial developmental stages (11, 29). Based on this, we would speculate that HVT vNr-13 also may contribute to the virus replication, vaccine responses, and even embryonic development. Furthermore, the chicken Nr-13 expressed at high levels in the embryonic bursa may function synergistically with HVT vNr-13 to support active replication through apoptosis inhibition. Thus, this may be the answer to why HVT has this vNr-13 homolog that is absent in other closely related avian alphaviruses.

The effect of HVT on cell viability further indicated that only a few HVT-infected cells were TUNEL positive (apoptotic). This is similar to the infections with other alphaherpesviruses, such as ILTV, HSV-1, HSV-2, and swine herpesvirus 1, which also demonstrated low levels of apoptosis (63–66). Furthermore, most of the TUNEL-positive cells appeared to be noninfected bystander cells present in the vicinity of HVT-infected cells. This picture is probably somewhat similar to that for the apoptotic local leukocytes that respond to productive infection, as observed in HSV-2 and HSV-1 infections *in vivo* (63, 65). Further investigation into the replication kinetics of HVT after *in ovo* or posthatch vaccination will be needed to know whether such bystander apoptotic cells have a role in HVT latency and/or reactivation.

Surprisingly, after 48 hpi, the viral titers in both HVT-Δ*vNr-13*- and wild-type-HVT-infected cells remained at similar levels, even though apoptotic cells were significantly higher in HVT-Δ*vNr-13*-infected cells than in wild-type-HVT-infected cells with high-PFU infection and vice versa with low-PFU infection (Fig. 9). Reasons for the continued production of viruses at comparable levels after 48 hpi despite the differences in the numbers of apoptotic cell populations are not fully clear. Although further, higher-PFU infection experiments might help in understanding the differences in growth kinetics between wild-type and mutant viruses, such experiments are difficult to perform due to the highly cell-associated nature of HVT. Furthermore, it is possible that there could be other antiapoptotic late viral genes that may be activated to inhibit apoptosis and increase virus replication after 48 hpi. It is known that herpesviruses such as MDV-1, HSV, and varicella zoster virus encode non-Bcl-2 oncoproteins with antiapoptotic properties (67). We have recently shown that Meq, a non-Bcl-2 oncoprotein of MDV, inhibits apoptosis and interacts with the chicken infectious anemia virus-encoded apoptotic protein apoptin (33, 68). Finally, the apoptotic regulation of vNr-13 appears to be biphasic, first at early time points in low-PFU infections and then at later time points in high-PFU infections, and it will be interesting to examine the roles of other viral/cellular factors involved in modulating apoptotic pathways, particularly at later time points of HVT infection.

The currently available apoptosis quantification techniques have limitations with reproducibility (69). In the past, some of studies have shown contrary results with the apoptotic cell regulation of Bcl-2 genes, such as Diva/Boo (25, 70). Thus, the IncuCyte S3-based investigation used in this study, as a label-free real-time monitoring of apoptotic cell kinetics, has the advantages of being fast, noninvasive, sensitive, and reproducible (8, 71–76). In fact, this method has been used for studies of real-time monitoring of cancer cell apoptosis, cell-to-cell interactions, drug design for evaluating apoptosis inducers and inhibitors, and viral replication kinetics (71, 73). With the high-throughput screening of more treatment groups and the increased sample size per group giving increased statistical power for the data (72, 74), the IncuCyte S3-based analysis of the kinetics of apoptosis used in this study strongly demonstrated the inhibitory role of *v-Nr-13* in apoptosis, especially with higher-PFU infections at later time points of HVT replication.

Development of antagonists as therapeutics to vBcl-2 proteins of EBV, KSHV, and other beta- and gammaherpesviruses is valuable to prevent viral infections. Up until now, most studies on therapeutics to vBcl-2 proteins have been based on *in vitro* experiments or animal models of different species. Currently, a more-physiological setting is required to elucidate the thermodynamics of vBcl-2 proteins and to understand their molecular mechanism in detail (41). As a naturally occurring nonpathogenic vaccine strain, HVT can be used as a very good model virus in cell cultures as well as embryonic avian tissues for better understanding of the molecular mechanisms and pathways of the Bcl-2 family of apoptotic modulators.

In conclusion, our results demonstrate that vNr-13 is a both structurally and functionally highly conserved, prosurvival Bcl-2 family protein. CRISPR/Cas9-based construction of HVT-Δ*vNr-13* indicated that the gene is not essential. However, the observations that (i) HVT-Δ*vNr-13* showed a lower viral yield at early time points than wild-type HVT, (ii) mitochondrial morphology was disrupted during HVT wild-type infection and substantially restored during HVT-Δ*vNr-13* infection, and (iii) the HVT vNr-13 homolog functions by inhibiting apoptosis, especially at later stages of virus replication, strongly suggested that it has an important role in sustaining virus replication by maintaining cells viable for long duration.

## MATERIALS AND METHODS

### Bioinformatics analysis of *vNr-13* gene.

The sequences of several mammalian and viral orthologs of Bcl-2 were retrieved from the NCBI database, and multiple-sequence alignments were performed using MEGA 6. 06. A phylogenetic tree was constructed using the maximum-likelihood method with substitution models and bootstrap values of the 1,000 replicates of the original data. The amino acid sequences of vNr-13 were submitted to the Iterative Threading ASSEmbly Refinement (I-TASSER) database online server (https://zhanglab.ccmb.med.umich.edu/I-TASSER/) to identify the predictive 3D structural models (40). The multiple aligned sequences were submitted to ESPript 3.0 (http://espript.ibcp.fr/ESPript/ESPript/index.php) analysis, using the I-TASSER database server-generated vNr-13 protein structure (PDB) as the template to predict secondary structures (24). The subcellular localization of the HVT vNr-13 protein was predicted using the available online algorithm DeepLoc-1.0 server (http://www.cbs.dtu.dk/services/DeepLoc/) (30).

### Cells, virus, and antibodies.

Primary chicken embryo fibroblasts (CEFs) were collected from 10-day-old embryonated specific-pathogen-free (SPF) eggs. CEFs were grown in M199 medium (Thermo Fisher Scientific) supplemented with 5% fetal calf serum (FCS) (Sigma), 1000 U/ml penicillin and 1 mg/ml streptomycin (Thermo Fisher Scientific), and 10% tryptose phosphate broth (Sigma). DF-1 cells, a continuous cell lines of EV-0 chicken embryo fibroblasts (77), were used in immunofluorescence (IF) and Western blot assays for analysis of the expression construct vNr-13. DF-1 cells were grown in high-glucose Dulbecco’s modified Eagle’s medium (DMEM) with GlutaMAX, supplemented with 10% fetal calf serum, 1000 U/ml penicillin, and 1 mg/ml streptomycin. The HVT FC126 strain (obtained from the Avian Disease and Oncology Laboratory [ADOL], East Lansing, MI, USA) was used to knock out both the HVT079 and HVT096 ORFs. The HVT FC126 strain was passaged on CEFs, and the third passage was used for the experiment. Mouse monoclonal anti-vNr-13 F878 EG2 (19) was used to label vNr-13, and chicken polyclonal anti-HVT antibodies were used to label HVT antigens (19). Alexa Fluor 488/568 goat anti-mouse IgG and Alexa Flour 488 goat anti-chicken IgG were the secondary antibodies used. Mouse monoclonal anti-α-tubulin was used to label tubulin protein as a cytosolic fraction control. Rabbit polyclonal anti-histone H3 was used to label histone H3 as a nuclear fraction control.

### Plasmids.

An expression construct of vNr-13 was generated using the pcDNA3.1(+) vector. For this, the full-length *vNr-13* was amplified by reverse transcription-PCR. Briefly, total RNA extracted from HVT-infected CEFs using the RNeasy minikit (Qiagen, Crawley, UK) was reverse transcribed using the Superscript III (Invitrogen) kit according to the manufacturer’s instructions. After denaturation of the reverse transcriptase at 70°C for 15 min, 5 μl of the reaction mixture was used in a 50-μl PCR mixture containing 10 mM deoxynucleoside triphosphate (dNTP), 10 μM each primer (Table 1), and 0.625 U of *Taq* DNA polymerase (Invitrogen). Insertion of *vNr-13* in the correct orientation in the recombinant pcDNA3.1-*v-Nr-13* construct was confirmed by sequence analysis. Plasmids pcDNA 3.1-flag-tagged-apoptin (encoding an inducer of apoptosis encoded by chicken infectious anemia virus) and pcDNA3.1-Meq (encoding an oncogenic protein encoded by Marek’s disease virus) were used as controls to monitor the apoptosis and have been described previously (33). Plasmid pX330A-1x2 was used for cloning of guide RNAs (gRNAs) for CRISPR/Cas9-based gene editing (see below).

### Analysis of expression of *vNr-13*.

DF-1 cells were plated in 24-well culture plates (1 × 10^5^) for immunofluorescence staining and in 6-well culture plates (1 × 10^6^) for Western blot analysis. For immunofluorescence (IF) staining, cells were plated onto glass coverslips. Cells were transfected (90 to 95% confluent at the time of transfection) with the pcDNA3.1-*vNr-13* construct using Lipofectamine 2000 reagent (Invitrogen, Karlsruhe, Germany). Mitochondria were stained (1 h, 37°C) with 200 nM MitoTracker Red CMXRos (Thermo Fisher). The endoplasmic reticulum (ER) was stained (30 min, 37°C) with 1 μM ER-Tracker Green (Bodipy FL Glibenclamide) (Thermo Fisher). Glibenclamide (glyburide) binds to the sulfonyl urea receptors of ATP-sensitive K^+^ channels, which are prominent on the ER. Transfected DF-1 cells then were fixed in 4% paraformaldehyde for 20 min at room temperature (RT) and permeabilized with 0.1% Triton X-100 (5 min, RT). After blocking in 5% bovine serum albumin (BSA) in phosphate-buffered saline (PBS) for 30 min, the cells were incubated (1 h, 37°C) with a 1:50 dilution of anti-vNr-13 F878 EG2 monoclonal antibody (19). After washing 3 times in PBS, cells were incubated (1 h, 37°C) with Alexa 488/568-conjugated goat anti-mouse antibody (1:200 in 5% BSA). After further washing in PBS, cells were stained (10 min, RT) with 4′,6′-diamidino-2-phenylindole (DAPI) (1:10,000) and viewed by using a Leica TCS SP2 (Wetzlar, Germany) microscope. A previously established Manders overlap coefficient method with a Coste significant level of 0.95 or above was followed to determine the colocalization using ImageJ 1.51j8 software (78).

For Western blot analysis, DF-1 cells transfected with pcDNA3.1-*vNr-13* were rinsed 48 h after transfection with ice-cold PBS and lysed in radioimmunoprecipitation assay (RIPA) buffer. Lysates were boiled with TruPAGE lithium dodecyl sulfate (LDS) sample buffer (Sigma) for 10 min at 95°C. The samples were separated on a 4 to 12% TruPAGE precast gel, and the resolved proteins were transferred onto polyvinylidene difluoride (PVDF) membranes. Immunoblots were blocked with 5% skimmed milk and then incubated (1 h, 37°C) with anti-vNr-13 F878 EG2 (1:20 dilution in 5% skimmed milk), after which blots were washed three times in Tris-buffered saline with 0.1% Tween 20 (TBST) and incubated (1 h, 37°C) with secondary antibody IRDye 680RD goat anti-mouse IgG (1:200 in 5% skimmed milk). Finally, after washing, blots were visualized using Odyssey Clx (Li-Cor).

The cytosolic and nuclear fractions were separated using a protocol based on an isolation kit (ab113474; Abcam). The purity of subcellular fractions was assayed with anti-α-tubulin or anti-histone H3 antibodies for the cytosolic or nuclear fractions, respectively. Western blots were analyzed as described above. Briefly, α-tubulin expression was examined using monoclonal mouse anti-α-tubulin as the primary antibody and IRDye 680RD goat anti-mouse IgG as the secondary antibody. Histone H3 was checked with rabbit polyclonal anti-histone H3 as the primary antibody and IRDye 800CW donkey anti-rabbit IgG as the secondary antibody.

### gRNAs for gene editing.

The guide RNA (gRNA) sequences were selected by identifying the 20-bp sequence directly upstream of any 5′-NGG sequence of exon 1 of both *vNr-13* ORFs HVT079 and HVT096, using the CRISPR guide RNA design online server (http://crispr.mit.edu/about). The gRNAs with highest predicted efficiency and lowest possible off-target cleavage sites were selected. Three gRNAs targeting in the 5′ and 3′ regions of exon 1 of the *vNr-13* gene were designed and cloned into the CRISPR/Cas9 vector pX330A-1x2 by introducing synthesized oligo-DNA primers corresponding to the target sequence into the BbsI restriction sites. The oligo-DNA primers are listed in Table 1.

### Generation of HVT-Δ*vNr-13* deletion mutant virus.

CEFs (1.3 × 10^6^) were plated in six-well culture plates and were 90 to 95% confluent at the time of transfection. At 12 h posttransfection, CEFs were infected with HVT at 100 PFU per well. The HVT inoculation dose is most accurately expressed as PFU because of the strict cell-associated phenotype of the virus, necessitating infection by inoculation of infected cells. Although the expression of MOI is not accurate because of the cell-associated nature of the virus and the difficulty to achieve synchronous infectivity, infection with 100 PFU of HVT corresponded to an MOI of approximately 0.00007. The infected CEFs were passaged 72 h later, and individual plaques were picked for further analysis.

### Characterization of HVT-Δ*vNr-13*.

CEFs were seeded in six-well culture plates and inoculated with wild-type HVT and purified mutant HVT-Δ*vNr-13*. The infected cells were harvested at 72 h after infection and cells lysed in 1× squishing buffer (10 mM Tris-HCl [pH 8.2], 25 mM NaCl, 1 mM EDTA, and 200 μg/ml proteinase K) at 65°C for 30 min. Proteinase K digestion was stopped by heating at 95°C for 2 min. the HVT079 and HVT096 *vNr-13* regions flanked with the 5′ and 3′ untranslated regions were amplified by PCR. The sequence-specific primers for the outside targets of the HVT079 and HVT096 *vNr-13* regions are listed in Table 1. The PCR products were purified, sequenced, and analyzed. In addition, IF staining was performed to confirm *vNr-13* deletion as described above. Briefly, wild-type HVT and mutant clones grown on CEF monolayers were examined for the expression of *vNr-13* using F878 EG2 as the primary antibody and Alexa 568-goat anti-mouse as the secondary antibody. HVT replication was detected using chicken polyclonal anti-HVT serum as the primary antibody and Alexa 488-goat anti-chicken as the secondary antibody.

### *In vitro* growth kinetics of wild-type HVT and HVT-Δ*vNr-13*.

The growth rates of wild-type HVT and HVT-Δ*vNr-13* were evaluated *in vitro* by determination of plaque counts and the average plaque areas on the confluent CEFs in six-well culture plates. Briefly, CEFs (1.3 × 10^6^) were plated in six-well culture plates, and wild-type HVT and HVT-Δ*vNr-13* virus at 100, 1,000 and 10,000 PFU per well, which correspond to MOIs of 0.00007, 0.0007, and 0.007, respectively, were inoculated in duplicates on confluent CEF monolayers. Infected CEFs from triplicate wells were trypsinized at 0, 12, 24, 48, 72, 96, and 120 h postinoculation (hpi) and titrated on fresh CEFs in 10-fold serial dilutions in six-well culture plates. Cultures were grown for 4 days before infected monolayers were washed, fixed, and enumerated by immunological staining using polyclonal HVT serum as described previously (79). Viral titers were log transformed prior to analysis.

The abilities of wild-type HVT and HVT-Δ*vNr-13* to produce cell-free virus from infected CEFs were also compared using protocols described previously (22). Briefly, supernatants were collected from cells inoculated with the wild-type HVT and HVT-Δ*vNr-13* viruses at 10,000 PFU/well (corresponding to an MOI of 0.007). Supernatants were centrifuged at 5,000 rpm for 10 min to remove any cells, and titers were determined at 0, 12, 24, 48, 72, 96, and 120 hpi.

### Measurement of virus plaque areas.

Plaque size measurements were performed on the immunologically stained wild-type HVT- and HVT-Δ*vNr-13*-infected CEF monolayers as described above. The average plaque area was measured as described previously (80). Digital images of over 60 individual plaques were captured using the EVOS digital microscope (Thermo Fisher Scientific, USA). The average plaque areas were measured using ImageJ 1.51j8 software by manually drawing the outline of each plaque. Plaque size measurements were performed in three biologically independent experiments.

### Analysis of mitochondrial morphology disturbance.

MitoTracker Red is extensively used to study the mitochondrial network morphology (14, 15, 17, 18). Thus, in this study MitoTracker Red was used to elucidate the mitochondrial network morphology by confocal microscopy. Disturbance to the mitochondrial network morphology was judged by the area of MitoTracker and distribution of intact mitochondrial network shapes. The average mitochondrial (MitoTracker) area was measured using ImageJ 1.51j8 software by manually drawing the outline of each Mito Tracker. When the outline was drawn, the intact tubular, branched, and punctate shapes were included for calculation. In the disturbed mitochondrial networks, the small and scattered punctate mitochondria at a distance were excluded for the calculation. The nuclear area was also excluded for the calculation. For elucidation of the intact mitochondrial network, the clear dispersed tubular and branched structures of more than 60% of Mito Tracker were considered intact mitochondria, whereas those of less than 60% were considered disturbed mitochondria. The mitochondrial morphology disturbance of vNr-13 and/or HVT-positive cells was determined in randomly selected fields of 100 cells. This measurement of mitochondrial morphology disturbance was performed in three independent experiments.

### Kinetics of apoptotic cells determined by high-throughput IncuCyte S3 real-time screening.

Inhibition of apoptosis in CEFs by the vNr-13 protein has been previously demonstrated (8). In our study, the main aim was to determine the kinetics of apoptosis following *vNr-13* expression, and we used the label-free high-throughput IncuCyte S3 (Sartorius AG, Gottingen, Germany) for real-time monitoring of apoptosis. Thus, to monitor the apoptosis kinetics of *vNr-13*, expression constructs of apoptin and Meq were used as apoptosis inducer and inhibitor controls, respectively (33). IncuCyte S3 is a fully automated phase-contrast microscope with an imaging system, designed to fit inside a cell culture incubator (71). First, DF-1 cells were transfected with vNr-13, apoptin, and Meq plasmids using the Lipofectamine 2000 reagent. The cell viability measurement in the transfected wells has a limitation due to a high percentage of apoptosis association with transfection reagents, such as Lipofectamine 2000 and Lipofectin (81, 82). Thus, to avoid experimental bias, the transfected cells were passaged after 48 h, and 5,000 and 10,000 transiently transfected cells were seeded in a 96-well plate (Corning) to avoid any transfection reagent-associated effects and to monitor the apoptotic cell kinetics up to 84 h. After overnight incubation, caspase 3/7 reagent (1:1,000) was added to the cells (Sartorius). Images were captured every 2 h for 84 h from four separate regions per well using a 10× objective. The green object count per well was quantified at each time point for vNr-13, apoptin, Meq, transfection control, and control cells.

The apoptotic cell kinetics of wild-type HVT and HVT-Δ*vNr-13* were determined with serum and under serum-free conditions using the caspase 3/7 apoptosis assay, and the experiment was monitored with the IncuCyte S3 as described above. Briefly, CEFs (4.5 × 10^4^) were plated in a 96-well culture plate (Nunc) and inoculated with 10, 50, and 100 PFU per well, which correspond to MOIs of 0.0002, 0.001, and 0.02, respectively, of wild-type HVT and HVT-Δ*vNr-13*. After virus inoculation, caspase 3/7 reagent (1:1,000; Sartorius) was added to the cells. Images were captured every 1 h for wells inoculated with 10 PFU and every 2 h for wells inoculated with 50 and 100 PFU for 50 h from four separate regions per well using a 10× objective. The green object count per well was quantified at each time point for wild-type HVT, HVT-Δ*vNr-13*, caspase 3/7 positive-control, and negative-control cells.

### Determination of cell viability by TUNEL assay.

The *In Situ* Cell Death Detection kit (fluorescein), based on terminal deoxynucleotidyltransferase-mediated dUTP-biotin nick end labeling (TUNEL) (Roche Diagnostics GmbH, Mannheim, Germany), was used to detect apoptotic cells in CEFs infected with wild-type HVT and HVT-Δ*vNr-13*. The test was performed according to the manufacturer’s guidelines. Briefly, CEFs (1.3 × 10^5^) were plated in 24-well culture plates onto glass coverslips, and wild-type HVT and HVT-Δ*vNr-13* were inoculated in triplicates on confluent CEFs at 10 PFU per well, which corresponds to an MOI of 0.00007. The IF test (see above) was performed using chicken polyclonal anti-HVT antibodies and Alexa Fluor 568 goat anti-chicken IgG. The percentage of TUNEL-positive cells was determined by manual counting ten randomly selected fields of 100 cells using a fluorescence microscope. Viability analysis was performed in three biologically independent experiments.

### Statistical Analysis.

IncuCyte S3 data were analyzed by one-way analysis of variance (ANOVA) with Tukey *post hoc* comparisons using GraphPad Prism version 7.01 (GraphPad Software, Inc., San Diego, CA). Growth kinetics of viral strains were evaluated by the Mann-Whitney U test. Mitochondrial morphology and TUNEL assay were evaluated by Student’s *t* test. Results were considered significantly different when the *P* value was <0.05. The results are shown as mean ± standard deviation (SD).

## Supplementary Material

Supplemental file 1

## References

[1] Kawamura H, King DJ Jr, Anderson DP 1969 A herpesvirus isolated from kidney cell culture of normal turkeys. Avian Dis 13:853–863. doi:10.2307/1588592. 4902778

[2] Witter RL, Nazerian K, Purchase HG, Burgoyne GH 1970 Isolation from turkeys of a cell-associated herpesvirus antigenically related to Marek’s disease virus. Am J Vet Res 31:525–538. 4314928

[3] Purchase HG, Okazaki W 1971 Effect of vaccination with herpesvirus of turkeys (HVT) on horizontal spread of Marek’s disease herpesvirus. Avian Dis 15:391–397. doi:10.2307/1588710. 4325826

[4] Kvansakul M, Caria S, Hinds MG 2017 The Bcl-2 family in host-virus interactions. Viruses 9:290. doi:10.3390/v9100290. PMC5691641 28984827

[5] Suraweera CD, Caria S, Jarva M, Hinds MG, Kvansakul M 2018 A structural investigation of NRZ mediated apoptosis regulation in zebrafish. Cell Death Dis 9:967. doi:10.1038/s41419-018-0992-0. 30237469 PMC6148235

[6] Polster BM, Pevsner J, Hardwick JM 2004 Viral Bcl-2 homologs and their role in virus replication and associated diseases. Biochim Biophys Acta 1644:211–227. doi:10.1016/j.bbamcr.2003.11.001. 14996505

[7] Gallo A, Lampe M, Gunther T, Brune W 2017 The viral Bcl-2 homologs of Kaposi’s sarcoma-associated herpesvirus and rhesus rhadinovirus share an essential role for viral replication. J Virol 91:e01875-16. doi:10.1128/JVI.01875-16. 28053098 PMC5331788

[8] Aouacheria A, Banyai M, Rigal D, Schmidt CJ, Gillet G 2003 Characterization of vnr-13, the first alphaherpesvirus gene of the bcl-2 family. Virology 316:256–266. doi:10.1016/j.virol.2003.08.014. 14644608

[9] Afonso CL, Tulman ER, Lu Z, Zsak L, Rock DL, Kutish GF 2001 The genome of turkey herpesvirus. J Virol 75:971–978. doi:10.1128/JVI.75.2.971-978.2001. 11134310 PMC113993

[10] Aouacheria A, Arnaud E, Venet S, Lalle P, Gouy M, Rigal D, Gillet G 2001 Nrh, a human homologue of Nr-13 associates with Bcl-Xs and is an inhibitor of apoptosis. Oncogene 20:5846–5855. doi:10.1038/sj.onc.1204740. 11593390

[11] Lee RM, Gillet G, Burnside J, Thomas SJ, Neiman P 1999 Role of Nr13 in regulation of programmed cell death in the bursa of Fabricius. Genes Dev 13:718–728. doi:10.1101/gad.13.6.718. 10090728 PMC316554

[12] Sheng ZH, Cai Q 2012 Mitochondrial transport in neurons: impact on synaptic homeostasis and neurodegeneration. Nat Rev Neurosci 13:77–93. doi:10.1038/nrn3156. 22218207 PMC4962561

[13] Takada S, Shirakata Y, Kaneniwa N, Koike K 1999 Association of hepatitis B virus X protein with mitochondria causes mitochondrial aggregation at the nuclear periphery, leading to cell death. Oncogene 18:6965–6973. doi:10.1038/sj.onc.1203188. 10597295

[14] Kramer T, Enquist LW 2012 Alphaherpesvirus infection disrupts mitochondrial transport in neurons. Cell Host Microbe 11:504–514. doi:10.1016/j.chom.2012.03.005. 22607803 PMC3358700

[15] Kim S, Kim HY, Lee S, Kim SW, Sohn S, Kim K, Cho H 2007 Hepatitis B virus x protein induces perinuclear mitochondrial clustering in microtubule- and dynein-dependent manners. J Virol 81:1714–1726. doi:10.1128/JVI.01863-06. 17151129 PMC1797565

[16] Cymerys J, Chodkowski M, Slonska A, Krzyzowska M, Banbura MW 2019 Disturbances of mitochondrial dynamics in cultured neurons infected with human herpesvirus type 1 and type 2. J Neurovirol 25:765–782. doi:10.1007/s13365-019-00762-x. 31161588 PMC6920257

[17] McCormick AL, Smith VL, Chow D, Mocarski ES 2003 Disruption of mitochondrial networks by the human cytomegalovirus UL37 gene product viral mitochondrion-localized inhibitor of apoptosis. J Virol 77:631–641. doi:10.1128/jvi.77.1.631-641.2003. 12477866 PMC140587

[18] Murata T, Goshima F, Daikoku T, Inagaki-Ohara K, Takakuwa H, Kato K, Nishiyama Y 2000 Mitochondrial distribution and function in herpes simplex virus-infected cells. J Gen Virol 81:401–406. doi:10.1099/0022-1317-81-2-401. 10644838

[19] Tang N, Zhang Y, Pedrera M, Chang P, Baigent S, Moffat K, Shen Z, Nair V, Yao Y 2018 A simple and rapid approach to develop recombinant avian herpesvirus vectored vaccines using CRISPR/Cas9 system. Vaccine 36:716–722. doi:10.1016/j.vaccine.2017.12.025. 29269155 PMC5783714

[20] Yuan M, Zhang W, Wang J, Al Yaghchi C, Ahmed J, Chard L, Lemoine NR, Wang Y 2015 Efficiently editing the vaccinia virus genome by using the CRISPR-Cas9 system. J Virol 89:5176–5179. doi:10.1128/JVI.00339-15. 25741005 PMC4403460

[21] Suenaga T, Kohyama M, Hirayasu K, Arase H 2014 Engineering large viral DNA genomes using the CRISPR-Cas9 system. Microbiol Immunol 58:513–522. doi:10.1111/1348-0421.12180. 25040500 PMC7168497

[22] Baigent SJ, Petherbridge LJ, Smith LP, Zhao Y, Chesters PM, Nair VK 2006 Herpesvirus of turkey reconstituted from bacterial artificial chromosome clones induces protection against Marek’s disease. J Gen Virol 87:769–776. doi:10.1099/vir.0.81498-0. 16528024

[23] Kingham BF, Zelnik V, Kopacek J, Majerciak V, Ney E, Schmidt CJ 2001 The genome of herpesvirus of turkeys: comparative analysis with Marek’s disease viruses. J Gen Virol 82:1123–1135. doi:10.1099/0022-1317-82-5-1123. 11297687

[24] Robert X, Gouet P 2014 Deciphering key features in protein structures with the new ENDscript server. Nucleic Acids Res 42:W320–W324. doi:10.1093/nar/gku316. 24753421 PMC4086106

[25] Song Q, Kuang Y, Dixit VM, Vincenz C 1999 Boo, a novel negative regulator of cell death, interacts with Apaf-1. EMBO J 18:167–178. doi:10.1093/emboj/18.1.167. 9878060 PMC1171112

[26] Kumar S, Stecher G, Tamura K 2016 MEGA7: Molecular Evolutionary Genetics Analysis version 7.0 for bigger datasets. Mol Biol Evol 33:1870–1874. doi:10.1093/molbev/msw054. 27004904 PMC8210823

[27] Zhang C, Freddolino PL, Zhang Y 2017 COFACTOR: improved protein function prediction by combining structure, sequence and protein-protein interaction information. Nucleic Acids Res 45:W291–W299. doi:10.1093/nar/gkx366. 28472402 PMC5793808

[28] Lu QL, Hanby AM, Nasser Hajibagheri MA, Gschmeissner SE, Lu PJ, Taylor-Papadimitriou J, Krajewski S, Reed JC, Wright NA 1994 Bcl-2 protein localizes to the chromosomes of mitotic nuclei and is correlated with the cell cycle in cultured epithelial cell lines. J Cell Sci 107:363–371. 8207068 10.1242/jcs.107.2.363

[29] Arnaud E, Ferri KF, Thibaut J, Haftek-Terreau Z, Aouacheria A, Le Guellec D, Lorca T, Gillet G 2006 The zebrafish bcl-2 homologue Nrz controls development during somitogenesis and gastrulation via apoptosis-dependent and -independent mechanisms. Cell Death Differ 13:1128–1137. doi:10.1038/sj.cdd.4401797. 16282981

[30] Almagro Armenteros JJ, Sonderby CK, Sonderby SK, Nielsen H, Winther O 2017 DeepLoc: prediction of protein subcellular localization using deep learning. Bioinformatics 33:3387–3395. doi:10.1093/bioinformatics/btx431. 29036616

[31] Yachida S, Kondo T, Hirai K, Izawa H, Mikami T 1986 Establishment of a variant type of turkey herpesvirus which releases cell-free virus into the culture medium in large quantities. Brief report. Arch Virol 91:183–192. doi:10.1007/bf01316738. 3753201

[32] Chodkowski M, Serafińska I, Brzezicka J, Golke A, Słońska A, Krzyżowska M, Orłowski P, Bąska P, Bańbura MW, Cymerys J 2018 Human herpesvirus type 1 and type 2 disrupt mitochondrial dynamics in human keratinocytes. Arch Virol 163:2663–2673. doi:10.1007/s00705-018-3890-y. 29872950 PMC6132932

[33] Brown AC, Reddy V, Lee J, Nair V 2018 Marek’s disease virus oncoprotein Meq physically interacts with the chicken infectious anemia virus-encoded apoptotic protein apoptin. Oncotarget 9:28910–28920. doi:10.18632/oncotarget.25628. 29988968 PMC6034753

[34] Okamoto T, Suzuki T, Kusakabe S, Tokunaga M, Hirano J, Miyata Y, Matsuura Y 2017 Regulation of apoptosis during flavivirus infection. Viruses 9:243. doi:10.3390/v9090243. PMC5618009 28846635

[35] Cuconati A, White E 2002 Viral homologs of BCL-2: role of apoptosis in the regulation of virus infection. Genes Dev 16:2465–2478. doi:10.1101/gad.1012702. 12368257

[36] Antoni BA, Sabbatini P, Rabson AB, White E 1995 Inhibition of apoptosis in human immunodeficiency virus-infected cells enhances virus production and facilitates persistent infection. J Virol 69:2384–2392. doi:10.1128/JVI.69.4.2384-2392.1995. 7884884 PMC188911

[37] Li H, Gao Q, Shao Y, Sun B, Wang F, Qiao Y, Wang N, Liu S 2018 Gallid herpesvirus 1 initiates apoptosis in uninfected cells through paracrine repression of p53. J Virol 92:e00529-18. doi:10.1128/JVI.00529-18. 29950417 PMC6146683

[38] Schumacher D, McKinney C, Kaufer BB, Osterrieder N 2008 Enzymatically inactive U(S)3 protein kinase of Marek’s disease virus (MDV) is capable of depolymerizing F-actin but results in accumulation of virions in perinuclear invaginations and reduced virus growth. Virology 375:37–47. doi:10.1016/j.virol.2008.01.026. 18304599 PMC2430872

[39] Haig DM 2001 Subversion and piracy: DNA viruses and immune evasion. Res Vet Sci 70:205–219. doi:10.1053/rvsc.2001.0462. 11676616

[40] Yang J, Yan R, Roy A, Xu D, Poisson J, Zhang Y 2015 The I-TASSER suite: protein structure and function prediction. Nat Methods 12:7–8. doi:10.1038/nmeth.3213. 25549265 PMC4428668

[41] Kvansakul M, Hinds MG 2013 Structural biology of the Bcl-2 family and its mimicry by viral proteins. Cell Death Dis 4:e909. doi:10.1038/cddis.2013.436. 24201808 PMC3847314

[42] Lee EF, Smith BJ, Horne WS, Mayer KN, Evangelista M, Colman PM, Gellman SH, Fairlie WD 2011 Structural basis of Bcl-xL recognition by a BH3-mimetic alpha/beta-peptide generated by sequence-based design. Chembiochem 12:2025–2032. doi:10.1002/cbic.201100314. 21744457 PMC3263372

[43] Suzuki M, Youle RJ, Tjandra N 2000 Structure of Bax: coregulation of dimer formation and intracellular localization. Cell 103:645–654. doi:10.1016/s0092-8674(00)00167-7. 11106734

[44] Clifton MC, Dranow DM, Leed A, Fulroth B, Fairman JW, Abendroth J, Atkins KA, Wallace E, Fan D, Xu G, Ni ZJ, Daniels D, Van Drie J, Wei G, Burgin AB, Golub TR, Hubbard BK, Serrano-Wu MH 2015 A maltose-binding protein fusion construct yields a robust crystallography platform for MCL1. PLoS One 10:e0125010. doi:10.1371/journal.pone.0125010. 25909780 PMC4409056

[45] Mandal PK, Ferreira LM, Collins R, Meissner TB, Boutwell CL, Friesen M, Vrbanac V, Garrison BS, Stortchevoi A, Bryder D, Musunuru K, Brand H, Tager AM, Allen TM, Talkowski ME, Rossi DJ, Cowan CA 2014 Efficient ablation of genes in human hematopoietic stem and effector cells using CRISPR/Cas9. Cell Stem Cell 15:643–652. doi:10.1016/j.stem.2014.10.004. 25517468 PMC4269831

[46] Mohr SE, Hu Y, Ewen-Campen B, Housden BE, Viswanatha R, Perrimon N 2016 CRISPR guide RNA design for research applications. FEBS J 283:3232–3238. doi:10.1111/febs.13777. 27276584 PMC5014588

[47] Liu Y, Cao Z, Wang Y, Guo Y, Xu P, Yuan P, Liu Z, He Y, Wei W 2018 Genome-wide screening for functional long noncoding RNAs in human cells by Cas9 targeting of splice sites. Nat Biotechnol 36:1203–1210. doi:10.1038/nbt.4283. 30395134

[48] Kosicki M, Tomberg K, Bradley A 2018 Repair of double-strand breaks induced by CRISPR-Cas9 leads to large deletions and complex rearrangements. Nat Biotechnol 36:765–771. doi:10.1038/nbt.4192. 30010673 PMC6390938

[49] Martinou JC, Youle RJ 2006 Which came first, the cytochrome c release or the mitochondrial fission? Cell Death Differ 13:1291–1295. doi:10.1038/sj.cdd.4401985. 16763618

[50] Rolland SG, Conradt B 2010 New role of the BCL2 family of proteins in the regulation of mitochondrial dynamics. Curr Opin Cell Biol 22:852–858. doi:10.1016/j.ceb.2010.07.014. 20729050 PMC2991415

[51] Norris KL, Youle RJ 2008 Cytomegalovirus proteins vMIA and m38.5 link mitochondrial morphogenesis to Bcl-2 family proteins. J Virol 82:6232–6243. doi:10.1128/JVI.02710-07. 18417572 PMC2447053

[52] Altmann M, Hammerschmidt W 2005 Epstein-Barr virus provides a new paradigm: a requirement for the immediate inhibition of apoptosis. PLoS Biol 3:e404. doi:10.1371/journal.pbio.0030404. 16277553 PMC1283332

[53] Gangappa S, van Dyk LF, Jewett TJ, Speck SH, Virgin HWt 2002 Identification of the in vivo role of a viral bcl-2. J Exp Med 195:931–940. doi:10.1084/jem.20011825. 11927636 PMC2193719

[54] Macen JL, Graham KA, Lee SF, Schreiber M, Boshkov LK, McFadden G 1996 Expression of the myxoma virus tumor necrosis factor receptor homologue and M11L genes is required to prevent virus-induced apoptosis in infected rabbit T lymphocytes. Virology 218:232–237. doi:10.1006/viro.1996.0183. 8615027

[55] Fleming P, Kvansakul M, Voigt V, Kile BT, Kluck RM, Huang DC, Degli-Esposti MA, Andoniou CE 2013 MCMV-mediated inhibition of the pro-apoptotic Bak protein is required for optimal in vivo replication. PLoS Pathog 9:e1003192. doi:10.1371/journal.ppat.1003192. 23468630 PMC3585157

[56] Jurak I, Schumacher U, Simic H, Voigt S, Brune W 2008 Murine cytomegalovirus m38.5 protein inhibits Bax-mediated cell death. J Virol 82:4812–4822. doi:10.1128/JVI.02570-07. 18321965 PMC2346748

[57] Pilder S, Logan J, Shenk T 1984 Deletion of the gene encoding the adenovirus 5 early region 1b 21,000-molecular-weight polypeptide leads to degradation of viral and host cell DNA. J Virol 52:664–671. doi:10.1128/JVI.52.2.664-671.1984. 6492257 PMC254571

[58] Liang Q, Chang B, Lee P, Brulois KF, Ge J, Shi M, Rodgers MA, Feng P, Oh BH, Liang C, Jung JU 2015 Identification of the essential role of viral Bcl-2 for Kaposi’s sarcoma-associated herpesvirus lytic replication. J Virol 89:5308–5317. doi:10.1128/JVI.00102-15. 25740994 PMC4442505

[59] Liang Q, Wei D, Chung B, Brulois KF, Guo C, Dong S, Gao SJ, Feng P, Liang C, Jung JU 2018 Novel role of vBcl2 in the virion assembly of Kaposi’s sarcoma-associated herpesvirus. J Virol 92:e00914-17. doi:10.1128/JVI.00914-17. 29167347 PMC5790944

[60] Sharma JM, Burmester BR 1982 Resistance to Marek’s disease at hatching in chickens vaccinated as embryos with the turkey herpesvirus. Avian Dis 26:134–149. doi:10.2307/1590032. 6284106

[61] Sharma JM, Witter RL 1983 Embryo vaccination against Marek’s disease with serotypes 1, 2 and 3 vaccines administered singly or in combination. Avian Dis 27:453–463. doi:10.2307/1590171. 6307248

[62] Sharma JM 1987 Delayed replication of Marek’s disease virus following in ovo inoculation during late stages of embryonal development. Avian Dis 31:570–576. doi:10.2307/1590742. 2823773

[63] Aleman N, Quiroga MI, Lopez-Pena M, Vazquez S, Guerrero FH, Nieto JM 2001 Induction and inhibition of apoptosis by pseudorabies virus in the trigeminal ganglion during acute infection of swine. J Virol 75:469–479. doi:10.1128/JVI.75.1.469-479.2001. 11119615 PMC113939

[64] Reddy VR, Steukers L, Li Y, Fuchs W, Vanderplasschen A, Nauwynck HJ 2014 Replication characteristics of infectious laryngotracheitis virus in the respiratory and conjunctival mucosa. Avian Pathol 43:450–457. doi:10.1080/03079457.2014.956285. 25144137

[65] Asano S, Honda T, Goshima F, Watanabe D, Miyake Y, Sugiura Y, Nishiyama Y 1999 US3 protein kinase of herpes simplex virus type 2 plays a role in protecting corneal epithelial cells from apoptosis in infected mice. J Gen Virol 80:51–56. doi:10.1099/0022-1317-80-1-51. 9934683

[66] Glorieux S, Bachert C, Favoreel HW, Vandekerckhove AP, Steukers L, Rekecki A, Van den Broeck W, Goossens J, Croubels S, Clayton RF, Nauwynck HJ 2011 Herpes simplex virus type 1 penetrates the basement membrane in human nasal respiratory mucosa. PLoS One 6:e22160. doi:10.1371/journal.pone.0022160. 21789229 PMC3137608

[67] Berkova Z, Wang S, Wise JF, Maeng H, Ji Y, Samaniego F 2009 Mechanism of Fas signaling regulation by human herpesvirus 8 K1 oncoprotein. J Natl Cancer Inst 101:399–411. doi:10.1093/jnci/djn516. 19276446 PMC2720696

[68] Brown AC, Baigent SJ, Smith LP, Chattoo JP, Petherbridge LJ, Hawes P, Allday MJ, Nair V 2006 Interaction of MEQ protein and C-terminal-binding protein is critical for induction of lymphomas by Marek’s disease virus. Proc Natl Acad Sci U S A 103:1687–1692. doi:10.1073/pnas.0507595103. 16446447 PMC1413633

[69] van de Schepop HA, de Jong JS, van Diest PJ, Baak JP 1996 Counting of apoptotic cells: a methodological study in invasive breast cancer. Clin Mol Pathol 49:M214–M217. doi:10.1136/mp.49.4.m214. 16696077 PMC408061

[70] Inohara N, Gourley TS, Carrio R, Muniz M, Merino J, Garcia I, Koseki T, Hu Y, Chen S, Nunez G 1998 Diva, a Bcl-2 homologue that binds directly to Apaf-1 and induces BH3-independent cell death. J Biol Chem 273:32479–32486. doi:10.1074/jbc.273.49.32479. 9829980

[71] Aftab O, Nazir M, Fryknas M, Hammerling U, Larsson R, Gustafsson MG 2014 Label free high throughput screening for apoptosis inducing chemicals using time-lapse microscopy signal processing. Apoptosis 19:1411–1418. doi:10.1007/s10495-014-1009-9. 24923770

[72] Hanson KM, Finkelstein JN 2019 An accessible and high-throughput strategy of continuously monitoring apoptosis by fluorescent detection of caspase activation. Anal Biochem 564–565:96–101. doi:10.1016/j.ab.2018.10.022. PMC6397776 30365977

[73] Chen J, Ribeiro B, Li H, Myer L, Chase P, Surti N, Lippy J, Zhang L, Cvijic ME 2018 Leveraging the IncuCyte technology for higher-throughput and automated chemotaxis assays for target validation and compound characterization. SLAS Discov 23:122–131. doi:10.1177/2472555217733437. 28957636

[74] Gupta S, Chan DW, Zaal KJ, Kaplan MJ 2018 A high-throughput real-time imaging technique to quantify NETosis and distinguish mechanisms of cell death in human neutrophils. J Immunol 200:869–879. doi:10.4049/jimmunol.1700905. 29196457 PMC5760330

[75] Johnston ST, Shah ET, Chopin LK, Sean McElwain DL, Simpson MJ 2015 Estimating cell diffusivity and cell proliferation rate by interpreting IncuCyte ZOOM assay data using the Fisher-Kolmogorov model. BMC Syst Biol 9:38. doi:10.1186/s12918-015-0182-y. 26188761 PMC4506581

[76] Cam M, Handke W, Picard-Maureau M, Brune W 2010 Cytomegaloviruses inhibit Bak- and Bax-mediated apoptosis with two separate viral proteins. Cell Death Differ 17:655–665. doi:10.1038/cdd.2009.147. 19816509

[77] Himly M, Foster DN, Bottoli I, Iacovoni JS, Vogt PK 1998 The DF-1 chicken fibroblast cell line: transformation induced by diverse oncogenes and cell death resulting from infection by avian leukosis viruses. Virology 248:295–304. doi:10.1006/viro.1998.9290. 9721238

[78] Dunn KW, Kamocka MM, McDonald JH 2011 A practical guide to evaluating colocalization in biological microscopy. Am J Physiol Cell Physiol 300:C723–C742. doi:10.1152/ajpcell.00462.2010. 21209361 PMC3074624

[79] Baigent SJ, Smith LP, Currie RJ, Nair VK 2005 Replication kinetics of Marek’s disease vaccine virus in feathers and lymphoid tissues using PCR and virus isolation. J Gen Virol 86:2989–2998. doi:10.1099/vir.0.81299-0. 16227220

[80] Jarosinski KW, Osterrieder N, Nair VK, Schat KA 2005 Attenuation of Marek’s disease virus by deletion of open reading frame RLORF4 but not RLORF5a. J Virol 79:11647–11659. doi:10.1128/JVI.79.18.11647-11659.2005. 16140742 PMC1212595

[81] Fiszer-Kierzkowska A, Vydra N, Wysocka-Wycisk A, Kronekova Z, Jarząb M, Lisowska KM, Krawczyk Z 2011 Liposome-based DNA carriers may induce cellular stress response and change gene expression pattern in transfected cells. BMC Mol Biol 12:27. doi:10.1186/1471-2199-12-27. 21663599 PMC3132718

[82] Wlodkowic D, Telford W, Skommer J, Darzynkiewicz Z 2011 Apoptosis and beyond: cytometry in studies of programmed cell death. Methods Cell Biol 103:55–98. doi:10.1016/B978-0-12-385493-3.00004-8. 21722800 PMC3263828

