# Supplementary material for: Novel Insights into the Roles of Bcl-2 Homolog Nr-13 (vNr-13) Encoded by Herpesvirus of Turkeys in the Virus Replication Cycle, Mitochondrial Networks, and Apoptosis Inhibition
Source: J Virol. 2020 May 4;94(10):e02049-19. doi: 10.1128/JVI.02049-19 (PMC7199394; doi:10.1128/JVI.02049-19)
Supplement: Supplemental file 1 [file JVI.02049-19-s0001.pdf]

Supplementary Materials

Supplementary Figures

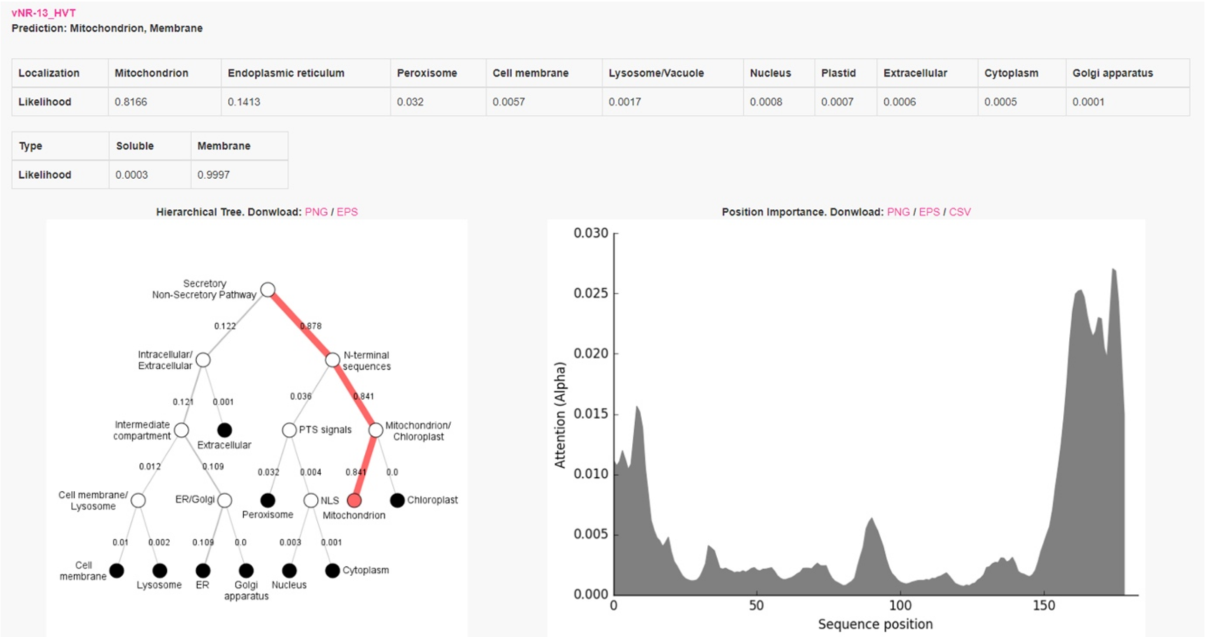

Supplementary figure 1. HVT *vNr-13* subcellular localization was predicted by online algorithm DeepLoc-1.0. Based on hierarchical tree, HVT *vNr-13* localizes to the mitochondria and endoplasmic reticulum membrane**.** Attention plot (alpha) suggests that specific interspersed region of HVT *vNr-13* between 150-179 amino acids involved in mitochondrial localization.
